# A heterodimeric glutathione *S*-transferase that stereospecifically breaks lignin's β(*R*)-aryl ether bond reveals the diversity of bacterial β-etherases

**DOI:** 10.1074/jbc.RA118.006548

**Published:** 2018-12-12

**Authors:** Wayne S. Kontur, Charles N. Olmsted, Larissa M. Yusko, Alyssa V. Niles, Kevin A. Walters, Emily T. Beebe, Kirk A. Vander Meulen, Steven D. Karlen, Daniel L. Gall, Daniel R. Noguera, Timothy J. Donohue

**Affiliations:** From the ‡ Wisconsin Energy Institute,; the § Department of Energy Great Lakes Bioenergy Research Center, and; the Departments of ¶ Biochemistry,; ‖ Civil and Environmental Engineering, and; ** Bacteriology, University of Wisconsin, Madison, Wisconsin 53706

**Keywords:** lignin degradation, stereoselectivity, protein evolution, bacterial metabolism, enzyme mutation, β-aryl ether bond, β-etherase, glutathione S-transferase, Novosphingobium aromaticivorans, sphingomonads

## Abstract

Lignin is a heterogeneous polymer of aromatic subunits that is a major component of lignocellulosic plant biomass. Understanding how microorganisms deconstruct lignin is important for understanding the global carbon cycle and could aid in developing systems for processing plant biomass into valuable commodities. Sphingomonad bacteria use stereospecific glutathione *S*-transferases (GSTs) called β-etherases to cleave the β-aryl ether (β-O-4) bond, the most common bond between aromatic subunits in lignin. Previously characterized bacterial β-etherases are homodimers that fall into two distinct GST subclasses: LigE homologues, which cleave the β(*R*) stereoisomer of the bond, and LigF homologues, which cleave the β(*S*) stereoisomer. Here, we report on a heterodimeric β-etherase (BaeAB) from the sphingomonad *Novosphingobium aromaticivorans* that stereospecifically cleaves the β(*R*)-aryl ether bond of the di-aromatic compound β-(2-methoxyphenoxy)-γ-hydroxypropiovanillone (MPHPV). BaeAB's subunits are phylogenetically distinct from each other and from other β-etherases, although they are evolutionarily related to LigF, despite the fact that BaeAB and LigF cleave different β-aryl ether bond stereoisomers. We identify amino acid residues in BaeAB's BaeA subunit important for substrate binding and catalysis, including an asparagine that is proposed to activate the GSH cofactor. We also show that BaeAB homologues from other sphingomonads can cleave β(*R*)-MPHPV and that they may be as common in bacteria as LigE homologues. Our results suggest that the ability to cleave the β-aryl ether bond arose independently at least twice in GSTs and that BaeAB homologues may be important for cleaving the β(*R*)-aryl ether bonds of lignin-derived oligomers in nature.

## Introduction

Lignocellulosic plant biomass is one of the most plentiful organic materials on Earth, so deciphering the mechanisms of its decomposition is an important part of understanding the global carbon cycle (1, 2). Lignin, which can make up as much as 30% of lignocellulosic biomass, is a heterogeneous polymer composed mainly of phenylpropanoid subunits linked together via several types of chemical bonds (3–5). Its abundance and composition make lignin a potential renewable raw material for the production of industrial chemicals, including low-molecular weight aromatic compounds, that are currently produced from petroleum (6, 7). However, the heterogeneous nature and chemical features of lignin make it difficult to depolymerize into defined products suitable for chemical upgrading. Indeed, many chemical methods for depolymerizing lignin produce heterogeneous mixtures of reactive radical compounds that can readily repolymerize (8). Thus, identifying and characterizing how microbes depolymerize lignin could both increase our understanding of the biological processes by which plant biomass is naturally recycled and aid in the development of industrial systems for the production of chemicals from this abundant renewable resource.

Biological degradation of the lignin polymer in nature is generally initiated by extracellular enzymes produced by fungi and some bacteria (9, 10). Sphingomonad bacteria use intracellular enzymes to mineralize the low-molecular weight aromatic oligomers and monomers produced in that initial stage of lignin degradation (11). Several sphingomonads are known or predicted to be capable of precise cleavage of many of the linkages between phenylpropanoid subunits in lignin (11, 12), including the β-aryl ether (β-O-4) bond, which can represent >50% of the total linkages (3). Among microbial lignin degraders, the enzymes used by sphingomonad bacteria to break the intersubunit bonds are of particular interest from an industrial standpoint, as they break the bonds in defined ways to generate predictable products that can readily be directed toward chemical upgrading. Indeed, *in vitro* systems using sphingomonad enzymes to break the β-aryl ether bonds of isolated lignin and/or model compounds have been reported (13–17).

Studies of *Sphingobium* sp. SYK-6 have shown that sphingomonads cleave the β-aryl ether bond of the di-aromatic model compound guaiacylglycerol-β-guaiacyl ether (GGE)^2^ in three steps (11). First, the α-hydroxyl of GGE is oxidized by NAD^+^-dependent dehydrogenases (LigD, LigN, LigL, and LigO in SYK-6 (18, 19)), which are reported to be stereoselective for the chirality of the bond's α-carbon, to generate the α-ketone, β-(2-methoxyphenoxy)-γ-hydroxypropiovanillone (MPHPV). Second, β-etherases (the glutathione *S*-transferases (GSTs) LigF, LigE, and LigP in SYK-6) replace the β-aryl ether bond of MPHPV with a β-thioether bond involving glutathione (GSH), producing guaiacol and the GSH conjugate, β-glutathionyl-γ-hydroxypropiovanillone (GS-HPV) (20, 21). Known bacterial β-etherases fall into two distinct GST subclasses and are strictly stereospecific for the chirality of the bond's β-carbon (22), with LigE homologues (which include LigP and which are similar to fungal FuA GSTs (23–25)) cleaving the β(*R*) isomer of the bond and LigF homologues (which are distinct in amino acid sequence and three-dimensional structure from known members of any previously established GST class (25)) cleaving the β(*S*) isomer. Finally, GSH lyases (the GSTs LigG (20) and SYK6GST_Nu_ (26) in SYK-6) remove the GSH moiety from GS-HPV and combine it with another GSH, producing hydroxypropiovanillone (HPV) and GSH disulfide (GSSG). LigG homologues, which share some features with Omega-class GSTs (27), are reported to preferentially cleave β(*R*)-GS-HPV, whereas the Nu-class GST_Nu_ homologues cleave both the β(*R*) and β(*S*) stereoisomers of GS-HPV with similar catalytic efficiencies (22, 26).

**Figure 1.**
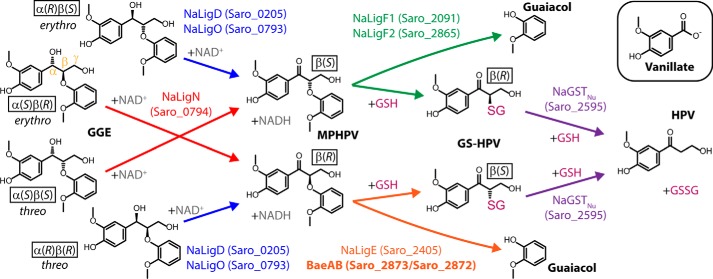
**Predicted *N. aromaticivorans* pathway for breaking the β-aryl ether (β-O-4) bond of GGE.** The dehydrogenases NaLigD, NaLigO, and NaLigN are predicted to oxidize GGE based on homology to enzymes in *Sphingobium* sp. SYK-6 (18) and the fact that their transcript abundances in *N. aromaticivorans* are increased by the presence of GGE (26). NaLigF1, NaLigF2, NaLigE, and NaGST_Nu_ have been characterized *in vitro* (26, 28, 29), and NaGST_Nu_ has been found to perform its reaction *in vivo* (26). This study reports the discovery and characterization of the stereospecific heterodimeric β-etherase BaeAB (in *boldface type*). The genes that code for the enzymes are shown in *parentheses*. The α, β, and γ carbons of the phenylpropanoid propyl chain are labeled in α(*S*)β(*R*)-GGE. Each chiral molecule is labeled with its stereoconfiguration. *Erythro*-GGE used in growth experiments consists of the α(*S*)β(*R*) and α(*R*)β(*S*) stereoisomers; *threo*-GGE consists of the α(*R*)β(*R*) and α(*S*)β(*S*) stereoisomers. *Inset*, vanillate, which was included in our growth experiments along with *erythro*-GGE (Fig. 2).

Although studies of *Sphingobium* sp. SYK-6 have provided significant understanding of the sphingomonad pathway for breaking the β-aryl ether bond, the details of this process are not identical in all sphingomonads. For example, *Novosphingobium aromaticivorans* DSM 12444 does not contain a stereoselective LigG homologue, but instead uses the Nu-class NaGST_Nu_ to remove the GSH moiety from both stereoisomers of GS-HPV (26) (Fig. 1). Here, we use mutant strains to show that *N. aromaticivorans* contains a β-etherase in addition to its LigE homologue (NaLigE) that stereospecifically breaks the β-aryl ether bond of β(*R*)-MPHPV, and we identify this β-etherase as a previously uncharacterized heterodimeric GST (named here BaeAB) encoded by Saro_2873 (*baeA*) and Saro_2872 (*baeB*). We identify amino acid residues in BaeAB's BaeA subunit involved in substrate binding and catalysis, including an asparagine, whose substitution with alanine dramatically affects catalysis, suggesting that the asparagine may be involved in activating the GSH cofactor. We also show that BaeAB has a catalytic efficiency similar to that of NaLigE. Notably, we find that the BaeA and BaeB polypeptides are phylogenetically distinct from each other and from the previously characterized β-etherases, although they are more phylogenetically similar to LigF homologues than to LigE homologues, even though BaeAB and LigF homologues cleave different stereoisomers of the β-aryl ether bond (Fig. 1). We also find that BaeAB homologues from the sphingomonads *Novosphingobium* sp. PP1Y and *Sphingobium xenophagum* can stereospecifically cleave β(*R*)-MPHPV and that BaeAB homologues are about as common in bacteria as LigE homologues, suggesting that BaeAB homologues may play an important role in breaking the β(*R*)-aryl ether bonds of lignin-derived oligomers in nature. Our results also suggest that the ability to cleave the β-aryl ether bond arose independently in the phylogenetically distinct LigF/BaeAB and FuA/LigE GST clusters, and although every individual β-etherase thus far characterized is specific for one of the stereoisomers of the bond, each cluster has members that cleave either the β(*R*) or β(*S*) stereoisomer.

## Results

### An N. aromaticivorans ΔNaLigE (Saro_2405) mutant can metabolize erythro-GGE

**Figure 2.**
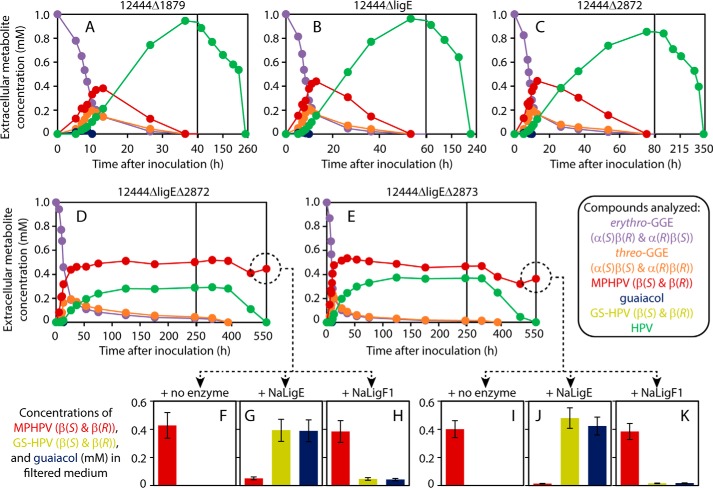
**Roles of *ligE*, Saro_2872, and Saro_2873 in GGE metabolism by *N. aromaticivorans*.** *Top* and *middle panels*, *N. aromaticivorans* strains 12444Δ1879 (parent; *A*), 12444ΔligE (*B*), 12444Δ2872 (*C*), 12444ΔligEΔ2872 (*D*), and 12444ΔligEΔ2873 (*E*) were grown in SMB containing 3 mm vanillate and 1 mm *erythro*-GGE. Extracellular aromatic compounds, *colored* according to the *key*, were analyzed at various time points (*A–E*). Extracellular concentrations of vanillate are not shown (in all cases, the vanillate provided was completely metabolized by 10–13 h). Note that *A–E* each use multiple *x* axis scales. *Bottom panels*, for the 12444ΔligEΔ2872 and 12444ΔligEΔ2873 cultures (*F–H* and *I–K*, respectively), filtered media from the final time points were combined with GSH and either H_2_O, recombinant NaLigE, or recombinant NaLigF1 to determine the stereoisomer(s) of MPHPV present in the media. *Error bars*, S.D.

Previous studies have shown that the LigE homologue from *N. aromaticivorans* (NaLigE, encoded by Saro_2405) stereospecifically cleaves the β-aryl ether bond of the β(*R*) stereoisomers of MPHPV and other di-aromatic compounds *in vitro* (28, 29). To investigate the *in vivo* role of NaLigE, we deleted Saro_2405 from *N. aromaticivorans* and grew the mutant strain (12444ΔligE), along with its parent strain (12444Δ1879), in medium containing vanillate and the di-aromatic compound *erythro*-GGE (consisting of the α(*S*)β(*R*) and α(*R*)β(*S*) stereoisomers; Fig. 1). As expected from a prior study (26), strain 12444Δ1879 completely consumed both the vanillate and the *erythro*-GGE, with the following aromatic intermediates of *erythro*-GGE metabolism (Fig. 1) transiently appearing in the medium: *threo*-GGE (likely formed from the back-reduction of MPHPV (26)), racemic (β(*R*) and β(*S*)) MPHPV, HPV, and a trace amount of guaiacol (Fig. 2*A*).

As NaLigE was the only known LigE homologue in *N. aromaticivorans* (29, 30) and as LigE homologues were the only sphingomonad β-etherases known to break the β(*R*)-aryl ether bond (22), we expected that strain 12444ΔligE would be unable to completely metabolize *erythro*-GGE. However, 12444ΔligE did completely metabolize the *erythro*-GGE (along with the vanillate), although the racemic MPHPV that transiently accumulated in the culture medium was metabolized more slowly by this strain than by strain 12444Δ1879 (Fig. 2, *A* and *B*). In addition, as the MPHPV disappeared from the 12444ΔligE culture medium, HPV transiently accumulated in the medium to a maximum concentration roughly equal to the initial *erythro*-GGE concentration (Fig. 2*B*), indicating that the GGE was completely converted into HPV (presumably via racemic MPHPV; Fig. 1). These results suggest that strain 12444ΔligE contains one or more enzymes that are capable of breaking the β-aryl ether bond of β(*R*)-MPHPV.

### The Saro_2872 and Saro_2873 gene products are required for cleavage of β(R)-MPHPV in N. aromaticivorans strain 12444ΔligE

As all known β-etherases are GSTs, we expected that another member of this enzyme superfamily was cleaving β(*R*)-MPHPV in strain 12444ΔligE. We investigated the predicted GST genes Saro_2872 and Saro_2873 because they are located in a gene cluster with the gene for the β-etherase NaLigF2 (Saro_2865) (29) (Fig. 1) and show a ∼3-fold increase in transcript abundance when cells are grown in the presence of GGE *versus* its absence.^3^ We separately deleted Saro_2872 and Saro_2873 from the genome of strain 12444ΔligE and grew the resulting strains (12444ΔligEΔ2872 and 12444ΔligEΔ2873, respectively) in the presence of vanillate and *erythro*-GGE. Both strains consumed the vanillate, but neither strain could fully metabolize the GGE (Fig. 2, *D* and *E*), instead accumulating extracellular MPHPV to a concentration roughly one-half of the culture's initial *erythro*-GGE concentration.

To identify the MPHPV stereoisomer(s) that remained in the media of the 12444ΔligEΔ2872 and 12444ΔligEΔ2873 cultures, the medium from each culture was filtered, and aliquots were combined with GSH and recombinant NaLigE or NaLigF1 (encoded by Saro_2091), which are known to stereospecifically cleave the β(*R*) or β(*S*) isomers of MPHPV, respectively (29) (Fig. 1). For each sample, the addition of NaLigE resulted in conversion of most of the MPHPV into GS-HPV and guaiacol (Fig. 2, *G* and *J*), and the addition of NaLigF1 resulted in conversion of <10% of the MPHPV into GS-HPV and guaiacol (Fig. 2, *H* and *K*). These results indicate that the MPHPV remaining in the 12444ΔligEΔ2872 and 12444ΔligEΔ2873 cultures was predominantly the β(*R*) isomer and thus that strain 12444ΔligE requires both Saro_2872 and Saro_2873 for complete metabolism of β(*R*)-MPHPV.

To test the role of Saro_2872 in cells containing a functional NaLigE protein, we deleted Saro_2872 from strain 12444Δ1879 to generate strain 12444Δ2872. When 12444Δ2872 was grown in the presence of vanillate and *erythro*-GGE, it completely metabolized both substrates, although it consumed the transiently accumulated extracellular MPHPV more slowly than 12444Δ1879 (Fig. 2, *A* and *C*), similar to the case for 12444ΔligE (Fig. 2*B*). Thus, it appeared that in a strain containing NaLigE, Saro_2872 is not needed for complete metabolism of MPHPV.

### The Saro_2873 (BaeA) and Saro_2872 (BaeB) polypeptides form a heterodimeric enzyme that stereospecifically cleaves β(R)-MPHPV

**Figure 3.**
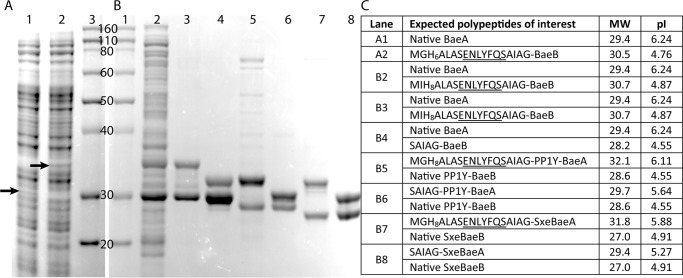
**SDS-PAGE of cell-free synthesized and recombinant BaeA (Saro_2873) and BaeB (Saro_2872) polypeptides.** Denatured proteins were separated on a NuPAGE 12% BisTris gel using NuPAGE MOPS SDS running buffer (Invitrogen). *A*, cell-free generated polypeptides. *B*, recombinant proteins. *Lane A1*, cell-free reaction mixture to produce the Saro_2873 polypeptide (BaeA; indicated by an *arrow*). *Lane A2*, cell-free reaction mixture to produce the Saro_2872 polypeptide containing an N-terminal His_6_ tag and intervening peptide linker (BaeB; indicated by an *arrow*). *Lanes A3* and *B1*, ladder, labeled with molecular weight in kDa (Novex Sharp pre-stained protein standard; Invitrogen). *Lane B2*, crude *E. coli* lysate containing recombinant BaeAB, with a His_8_ tag and intervening peptide linker on the BaeB subunit N terminus. *Lane B3*, elution from a Ni^2+^-NTA column using imidazole after passing the crude *E. coli* lysate from *lane B2* through the column. *Lane B4*, flow-through from a Ni^2+^-NTA column after cleaving the His_8_ tag off the protein in *lane B3. Lane B5*, elution from a Ni^2+^-NTA column using imidazole after passing crude *E. coli* lysate containing recombinant PP1Y-BaeAB with a His_8_ tag and intervening peptide linker on the PP1Y-BaeA N terminus through the column. *Lane B6*, flow-through from a Ni^2+^-NTA column after cleaving the His_8_ tag off the protein in *lane B5. Lane B7*, elution from a Ni^2+^-NTA column using imidazole after passing crude *E. coli* lysate containing recombinant SxeBaeAB with a His_8_ tag and intervening peptide linker on the SxeBaeA N terminus through the column. *Lane B8*, flow-through from a Ni^2+^-NTA column after cleaving the His_8_ tag off the protein in *lane B7*. The denatured polypeptides did not all run at their expected molecular weights, possibly due to differences in pI values. *C*, predicted molecular weights (*MW*) (kDa) and isoelectric points (pI) of polypeptides analyzed via SDS-PAGE, calculated using ExPASy (52). TEV protease recognition sites are *underlined* in the sequences.

**Figure 4.**
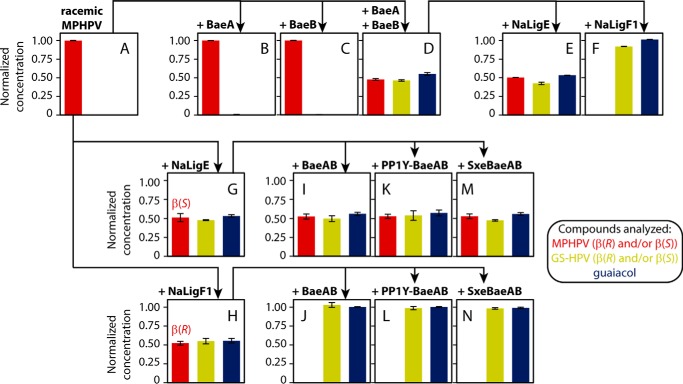
**Cleavage of MPHPV by cell-free synthesized Saro_2873 (BaeA) and Saro_2872 (BaeB) polypeptides, and by recombinant BaeAB from *N. aromaticivorans*, *Novosphingobium* sp. PP1Y, and *S. xenophagum*.** *Top row*, racemic (β(*R*) and β(*S*)) MPHPV (*A*) was mixed with cell-free synthesized BaeA (Saro_2873) (*B*) or BaeB (Saro_2872) (*C*) individually or a mixture of both BaeA and BaeB (*D*). The reaction containing both BaeA and BaeB was split and combined with either NaLigE (*E*) or NaLigF1 (*F*), which are stereospecific for β(*R*)- or β(*S*)-MPHPV, respectively. *Middle* and *bottom rows*, racemic MPHPV (*A*) was mixed with either NaLigE (*G*) or NaLigF1 (*H*) to generate enantiopure β(*S*)- or β(*R*)-MPHPV, respectively. These samples were then combined with recombinant BaeAB from *N. aromaticivorans* (*I* and *J*), *Novosphingobium* sp. PP1Y (PP1Y-BaeAB; *K* and *L*), or *S. xenophagum* (SxeBaeAB; *M* and *N*). Compounds analyzed are *colored* according to the *key*. All reactions contained ≥5 mm GSH. Normalized concentrations are the concentrations of the various compounds analyzed divided by the initial racemic MPHPV concentration used in each assay. Representative HPLC traces from these experiments are shown in Figs. S1, S3, and S8. *Error bars*, S.D.

As our mutant results suggested that the Saro_2873 and Saro_2872 gene products contribute to MPHPV cleavage in *N. aromaticivorans*, we sought to test whether they performed this reaction *in vitro*. Attempts to separately produce the individual Saro_2873 and Saro_2872 polypeptides from recombinant *Escherichia coli* cultures were unsuccessful, but we were able to separately produce each polypeptide using a cell-free system (Fig. 3*A*). When the Saro_2873 or Saro_2872 polypeptides were individually incubated with racemic (β(*R*) and β(*S*)) MPHPV and GSH, only a trace amount of GS-HPV was observed (representing <1% conversion of the MPHPV present in the reaction) (Fig. 4 (*A–C*) and Fig. S1 (*A–C*)), even after several days. (Under these same reaction conditions, the β-etherases NaLigE and NaLigF1 are active (Fig. 4, *G* and *H*).)

The apparent inability of the individual Saro_2873 and Saro_2872 polypeptides to cleave MPHPV, combined with our observation that loss of either Saro_2873 or Saro_2872 rendered 12444ΔligE unable to metabolize β(*R*)-MPHPV (Fig. 2, *D* and *E*) and the fact that Saro_2873 and Saro_2872 overlap in the *N. aromaticivorans* genome (Fig. S2) suggested that the polypeptides may form a heterooligomeric complex. Indeed, when we repeated our enzyme assay using a mixture of the Saro_2873 and Saro_2872 polypeptides, half of the racemic MPHPV substrate was converted into GS-HPV and guaiacol, suggesting that the individual polypeptides formed a catalytically active enzyme complex in the reaction mixture (Fig. 4*D* and Fig. S1*D*). To determine which stereoisomer(s) of MPHPV remained after this reaction, we added recombinant NaLigE or NaLigF1 to aliquots of the reaction mixture. Upon the addition of NaLigE, there were no detectable changes in the amounts of MPHPV, GS-HPV, or guaiacol (Fig. 4*E* and Fig. S1*E*). In contrast, upon the addition of NaLigF1, the MPHPV was completely converted into GS-HPV and guaiacol (Fig. 4*F* and Fig. S1*F*), indicating that the MPHPV that remained in the reaction mixture that contained both the Saro_2873 and Saro_2872 polypeptides was the β(*S*) stereoisomer, consistent with our hypothesis that the Saro_2873–Saro_2872 enzyme complex cleaves β(*R*)-MPHPV.

**Figure 5.**
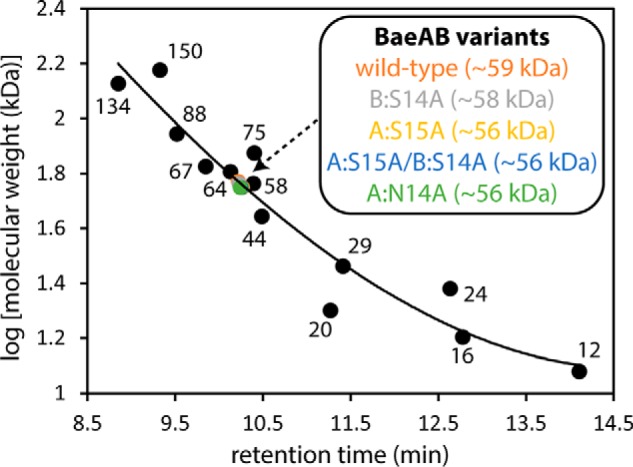
**Gel permeation chromatography of BaeAB.** WT and variant BaeAB proteins (Saro_2873–Saro_2872 polypeptide complexes) from *N. aromaticivorans* were analyzed under nondenaturing conditions, along with proteins of known molecular weights (labeled with MW in kDa): bovine heart cytochrome *c* monomer and dimer (12 and 24 kDa), RNase A (16 kDa), *G. max* trypsin inhibitor (20 kDa), bovine erythrocyte carbonic anhydrase monomer and dimer (29 and 58 kDa), ovalbumin monomer and dimer (44 and 88 kDa), NaGST_Nu_ dimer (64 kDa), BSA monomer and dimer (67 and 134 kDa), and conalbumin monomer and dimer (75 and 150 kDa). The data for the proteins of known molecular weight were fit to a cubic curve, which was used to calculate the apparent molecular weights of WT BaeAB and the BaeAB variants. The individual BaeA and BaeB polypeptides are predicted to be ∼29 kDa (Fig. 3).

To further test this hypothesis, we cloned Saro_2873 and Saro_2872 into a single expression vector and observed that significant amounts of soluble recombinant protein were produced in *E. coli* harboring this plasmid (Fig. 3*B*, *lane B2*). This suggested that our difficulty in expressing each of the polypeptides individually in *E. coli* may have been because they could not fold correctly in the absence of the other polypeptide and thus were subject to proteolytic cleavage. In addition, despite the fact that a His_8_ tag was attached only to the Saro_2872 polypeptide (with an intervening peptide containing a tobacco etch virus (TEV) protease recognition site), the protein purified from *E. coli* lysate using Ni^2+^-chelate chromatography was composed of two polypeptides, with apparent molecular weights as assessed by SDS-PAGE similar to the expected molecular weights of the individual His_8_-Saro_2872 and Saro_2873 polypeptides (∼30 kDa) (Fig. 3*B*, *lane B3*). When the His_8_ tag was cleaved from the protein using TEV protease, neither of the polypeptides bound to Ni^2+^-chelate resin, and one of the polypeptides decreased in molecular weight, as assessed by SDS-PAGE (Fig. 3*B*, *lane B4*). These results supported the idea that the polypeptides bound to each other to form an enzyme complex. We also analyzed the purified recombinant protein complex by gel permeation chromatography (GPC) and found that it eluted as a single peak with an apparent molecular weight of ∼59 kDa (Fig. 5). This suggested that the complex was a heterodimer, consistent with the fact that previously characterized GSTs generally function as (homo- or hetero-) dimers (31, 32).

We incubated the purified recombinant heterodimer with enzymatically generated enantiopure samples of β(*S*)- or β(*R*)-MPHPV and GSH (Fig. 4 (*G* and *H*) and Fig. S3 (*A–C*)). Consistent with our results with the cell-free generated polypeptides, the recombinant enzyme had no effect on β(*S*)-MPHPV and converted β(*R*)-MPHPV into GS-HPV and guaiacol (Fig. 4 (*I* and *J*) and Fig. S3 (*D* and *E*)). Thus, we concluded that the Saro_2873 and Saro_2872 polypeptides (BaeA and BaeB, respectively) represent subunits of a newly discovered β(*R*)-aryl ether bond cleaving heterodimeric GST (named here BaeAB).

### Amino acid substitutions in the BaeA subunit affect catalysis by the BaeAB heterodimer, but an analogous substitution in the BaeB subunit has no effect on catalysis

We sought to gain insight into the relative contributions of the BaeA and BaeB subunits to the catalytic activity of BaeAB by modifying the predicted active-site residues in the individual subunits. Each of the variant BaeAB proteins described below were expressed in and purified from *E. coli*, and SDS-PAGE analysis indicated that each one contained both a BaeA and BaeB subunit. In addition, each variant enzyme eluted as a single peak in GPC analysis, with an apparent molecular weight that was indistinguishable from that of the WT enzyme (Fig. 5), suggesting that any impact of the amino acid substitutions on enzyme activity is not due to large-scale defects in folding or changes in the oligomeric state of the enzyme.

**Figure 6.**
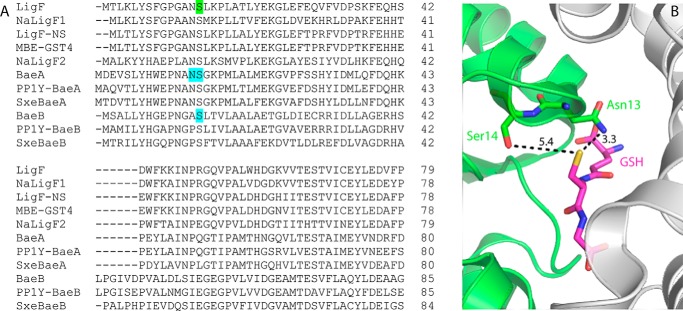
**Comparison of LigF homologues with BaeA (Saro_2873) and BaeB (Saro_2872) homologues.** *A*, alignment of LigF homologues previously shown to cleave β(*S*)-MPHPV, along with BaeA and BaeB homologues from *N. aromaticivorans*, *Novosphingobium* sp. PP1Y, and *S. xenophagum* shown in this study to cleave β(*R*)-MPHPV. The alignment is shown for the N-terminal thioredoxin domain; a complete sequence alignment is shown in Fig. S4. Proteins analyzed are NaLigF1 (Saro_2091; WP_041551020.1), NaLigF2 (Saro_2865; WP_052241956.1), BaeA (Saro_2873; WP_011446513.1), and BaeB (Saro_2872; WP_011446512.1) from *N. aromaticivorans* (29); LigF (SLG_08650; WP_014075191.1) from *Sphingobium* sp. SYK-6 (20); LigF-Ns (PP1Y_AT11660; WP_013832480.1), PP1Y-BaeA (PP1Y_AT11532; WP_013832467.1), and PP1Y-BaeB (PP1Y_AT11540; WP_013832468.1) from *Novosphingobium* sp. PP1Y (28); MBE-GST4 (MBENS4_2528; WP_039391123.1) from *Novosphingobium* sp. MBES04 (30); and SxeBaeA (SX1_RS06450; WP_019052344.1) and SxeBaeB (SX1_RS06455; WP_019052345.1) from *S. xenophagum*. Protein sequences were aligned using Clustal Omega (53). The serine residue (Ser^14^) previously found to be involved in catalysis in LigF (25) is *highlighted* in *green*. The BaeA and BaeB residues substituted by alanine in this study are highlighted in *blue. B*, active site of LigF from *Sphingobium* sp. SYK-6 (Protein Data Bank code 4XT0). Carbon atoms for the LigF N-terminal thioredoxin domain and for the bound GSH are *colored green* and *magenta*, respectively. Oxygen, nitrogen, and sulfur atoms are *colored red*, *blue*, and *yellow*, respectively. The LigF C-terminal GST domain is *colored gray*. Distances in Angstroms are shown for the GSH thiol to the side-chain hydroxyl of Ser^14^ and to the side-chain amide nitrogen of Asn^13^.

As attempts to determine the crystal structure of BaeAB have thus far been unsuccessful, we ran a search of the SWISS-MODEL template library of structurally characterized proteins (33) and found that BaeA and BaeB are each most similar in amino acid sequence to LigF from *Sphingobium* sp. SYK-6 (SLG_08650; WP_014075191.1; Protein Data Bank code 4XT0) (even though LigF and BaeAB cleave different stereoisomers of the β-aryl ether bond). We aligned the amino acid sequences of BaeA and BaeB with that of LigF to formulate predictions for which of BaeAB's residues may be involved in catalysis (Fig. 6*A*). LigF contains an active-site serine residue (Ser^14^) previously found to be important for cleaving the MPHPV analogue β(*S*)-(1′-formyl-3′-methoxyphenoxy)-γ-hydroxypropioveratrone (25). This serine residue is conserved in all LigF homologues previously shown to have β-etherase activity and in both BaeA (Ser^15^) and BaeB (Ser^14^) (Fig. 6*A* and Fig. S4). To test the roles of these serine residues in BaeAB, we substituted them with alanines individually (A:S15A and B:S14A) and together (A:S15A/B:S14A) and investigated the *in vitro* kinetics of cleaving the β-aryl ether bond of β(*R*)-MPHPV by WT BaeAB and the variant enzymes (Fig. S5). We found that the variant in which BaeA Ser^15^ was changed to an alanine (A:S15A) had a *k*_cat_ value similar to that of WT BaeAB and a *K_m_* value that was 5–6-fold higher than that of WT BaeAB (Table 1), suggesting that the side-chain hydroxyl of BaeA Ser^15^ is involved in substrate binding, but not in catalytic turnover of BaeAB. The variant in which BaeB Ser^14^ was changed to an alanine (B:S14A) had the same *k*_cat_ and *K_m_* values as WT BaeAB (Table 1), suggesting that BaeB Ser^14^ is not involved in catalytic turnover or substrate binding by BaeAB. The variant in which both BaeA Ser^15^ and BaeB Ser^14^ were changed to alanines (A:S15A/B:S14A) behaved the same as the A:S15A variant (Table 1), supporting the importance of BaeA Ser^15^ in substrate binding and the noninvolvement of BaeB Ser^14^ in catalysis by BaeAB.

**Table 1:** **Kinetic parameters for the enzymatic conversion of β(***R***)–MPHPV into GS-HPV** Parameters are from non-linear least-squares best fits of the Michaelis–Menten equation to plots of initial rate ([GS-HPV] formed/s) *versus* β(*R*)-MPHPV concentration (Fig. S5). For the BaeAB enzymes, the concentrations of the heterodimers were used in the equation; for NaLigE, the concentration of the individual polypeptide subunits of the homodimer was used.

Protein	*k*_cat_	*K_m_*	*k*_cat_/*K_m_*
	*s*^−*1*^	μ*m*	*mm*^−*1*^ *s*^−*1*^
BaeAB	2.9 ± 0.3	20 ± 3	150 ± 30
BaeAB (A:S15A)	2.4 ± 0.3	100 ± 20	23 ± 6
BaeAB (B:S14A)	2.8 ± 0.2	22 ± 3	130 ± 20
BaeAB (A:S15A/B:S14A)	2.8 ± 0.5	120 ± 30	23 ± 8
BaeAB (A:N14A)	0.14 ± 0.02	250 ± 60	0.5 ± 0.2
NaLigE	0.68 ± 0.06	7 ± 1	100 ± 20

The three-dimensional structure of LigF shows that the side-chain amide nitrogen of its Asn^13^ is within hydrogen-bonding distance (3.3 Å) of the bound GSH thiol (Fig. 6*B*), suggesting that the asparagine may be involved in activating the GSH thiol (GST reactivity typically involves activation of a GSH molecule by stabilizing it in its thiolate form through hydrogen-bonding with an enzyme active-site residue (34)). We found that an analogous asparagine is present in all of the LigF homologues previously shown to have β-etherase activity and in BaeA (Asn^14^), although BaeB has an alanine in this position (Fig. 6*A*). Active-site asparagine residues are known (35, 36) or predicted (26) to be involved in catalysis by some other GSTs. When we replaced Asn^14^ in BaeA with an alanine, we found that the resulting variant (A:N14A) had a *k*_cat_ value ∼20-fold lower and a *K_m_* value ∼12.5-fold higher than WT BaeAB (Table 1), suggesting that this asparagine side chain is critical for both substrate binding and turnover in BaeAB. This large negative effect of substituting a single amino acid residue in BaeA on catalysis by BaeAB suggests that BaeA plays a major role in the heterodimer's reactivity. Indeed, if the BaeA subunit were completely inactivated in the A:N14A variant, a comparison of *k_cat_*/*K_m_* values for WT BaeAB and the A:N14A variant suggests that BaeB has a catalytic efficiency with β(*R*)-MPHPV as substrate that is <0.3% of BaeA's catalytic efficiency. In addition, if the GSH binding pocket in BaeB is structurally similar to that of LigF, our amino acid sequence alignment predicts that there may not be any polar residues in BaeB other than Ser^14^ in a position to activate the thiol of a GSH molecule bound in BaeB (Fig. 6*A*), which would be consistent with BaeB having a lower catalytic activity than BaeA.

### Comparing BaeAB and NaLigE catalysis

We sought to compare the catalytic abilities of BaeAB and NaLigE, the two *N. aromaticivorans* enzymes now known to cleave the β(*R*)-aryl ether bond. For this, we tested purified recombinant NaLigE for cleavage of β(*R*)-MPHPV *in vitro* under conditions identical to those used to test BaeAB (Fig. S5) and found that NaLigE had a ∼4-fold lower *k*_cat_ value and a ∼3-fold lower apparent *K_m_* value than BaeAB, leading to a measured catalytic efficiency (*k*_cat_/*K_m_*) for NaLigE that is slightly lower than that of BaeAB (Table 1). Thus, the newly discovered heterodimeric β-etherase BaeAB is catalytically comparable with, or even more efficient than, NaLigE at cleaving β(*R*)-MPHPV.

### The stereospecificity of BaeAB is unexpected from its phylogeny

**Figure 7.**
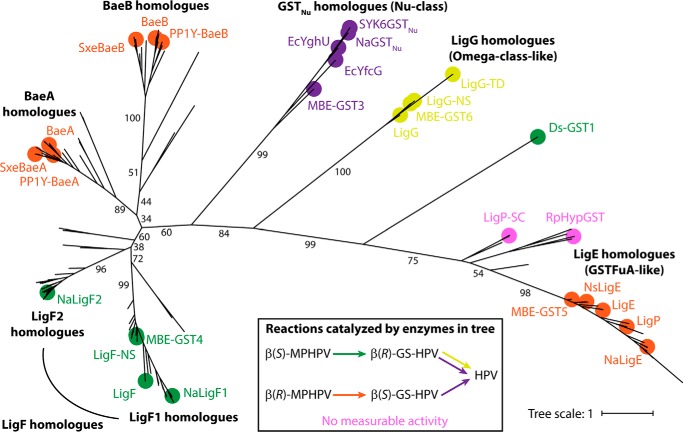
**Phylogenetic tree of GSTs known or predicted to catalyze reactions involved in the sphingomonad pathway for breaking the β-aryl ether bond.** For proteins that have been tested for activity, nodes are marked with a *circle color-coded* for the observed catalytic activities (shown in the *key*). Proteins shown to catalyze a reaction include the LigF, BaeA, and BaeB homologues analyzed in Fig. 6, as well as NaLigE (Saro_2405; WP_011446047.1) and NaGST_Nu_ (Saro_2595; WP_011446237.1) from *N. aromaticivorans* (26, 28, 29); LigE (SLG_08660; WP_014075192.1), LigP (SLG_32600; WP_014077574.1), LigG (SLG_08670; WP_041392591.1), and SYK6GST_Nu_ (SLG_04120; WP_014074739.1) from *Sphingobium* sp. SYK-6 (20, 26, 28, 29, 54); MBE-GST3 (MBENS4_2527; WP_039391121.1), MBE-GST5 (MBENS4_2529; WP_039391125.1), and MBE-GST6 (MBENS4_2530; WP_052322378.1) from *Novosphingobium* sp. MBES04 (30); NsLigE (PP1Y_AT11664; WP_013832481.1) and LigG-NS (PP1Y_AT11674; WP_041558818.1) from *Novosphingobium* sp. PP1Y (28, 29, 54); LigG-TD (Tbd_1050; WP_011311562.1) from *Thiobacillus denitrificans* (54); EcYghU (ECDH1ME8569_2889; BAJ44745.1) and EcYfcG (ECDH1ME8569_2240; BAJ44096.1) from *E. coli* (26); and Ds-GST1 (XP_007363869.1) from *D. squalens* (39). Tested proteins for which no measurable activity was observed are LigP-SC (WP_012238501.1) from *S. cellulosum* (28) and RpHypGST (RPA4340; WP_027276361.1) from *R. palustris* (29). Unmarked nodes represent proteins that have not been tested for activity (for a full list, see Table S3). Selected bootstrap values are displayed. Class names are shown in *parentheses* for clusters previously proposed to be members of established GST classes (see “Discussion”). A Newick file for the phylogenetic tree is included in the supporting information.

The five bacterial β-etherases that have previously been shown to break the β(*R*)-aryl ether bond (LigE homologues; Fig. S6) share >59% amino acid sequence identity with each other, and the five bacterial β-etherases that have previously been shown to break the β(*S*)-aryl ether bond (LigF homologues; Fig. 6*A* and Fig. S6) share >39% amino acid sequence identity with each other (four of these LigF homologues share >60% sequence identity between them, but NaLigF2 is only ∼40% identical to the others). In contrast, the LigE and LigF homologues share <22% amino acid sequence identity between them (Fig. S6). An amino acid sequence analysis of all of the GSTs involved in the sphingomonad pathway for breaking the β-aryl ether bond shows that the LigF and LigE homologues form separate phylogenetic clades (Fig. 7), consistent with the low sequence identity between them. In addition, the LigF homologues are split into two related, but distinct, subclades, which we refer to as LigF1 and LigF2 homologues (Fig. 7).

BaeA and BaeB share ∼24% amino acid sequence identity with each other (Fig. 6*A* and Fig. S6) and fall into separate phylogenetic clades (Fig. 7). They are also both distinct in amino acid sequence from other β-etherases (Fig. S6): BaeA is <36% and <17% identical to the LigF and LigE homologues, respectively, and BaeB is <30% and <18% identical to the LigF and LigE homologues, respectively. However, BaeA and BaeB are both phylogenetically closer to the LigF homologues than to the LigE homologues (Fig. 7), despite the fact that BaeAB cleaves the β(*R*)-aryl ether bond, and LigF homologues cleave the β(*S*)-aryl ether bond.

### Distributions of BaeAB and LigE homologues among bacteria

Based on a BLASTp search of the NCBI database (performed in October 2018), the genomes of 43 species were predicted to contain genes for homologues of both BaeA and BaeB (using an amino acid sequence identity >50% and a bit score >200 as criteria), and 48 species were predicted to contain at least one gene for a LigE homologue (six of these contain genes for two LigE homologues) (Table S1). Of these bacteria, 22 (including *N. aromaticivorans*, *Novosphingobium* sp. MBES04, and *Novosphingobium* sp. SG61-1L, which are known to metabolize *erythro*-GGE (19, 26, 30)) were predicted to encode both a BaeAB and a LigE homologue. These results suggest that BaeAB homologues may be about as common in bacteria as LigE homologues.

In all of the bacteria predicted to contain a BaeAB homologue, the BaeA structural gene is directly upstream of the gene for BaeB, and in all but one case, the genes overlap (Table S1), an arrangement that is often seen for polypeptides that form a heterooligomeric complex, further supporting the prediction that these species contain active BaeAB heterodimers. Interestingly, there is greater amino acid sequence identity among the BaeA homologues than among the BaeB homologues (Fig. S7), which is consistent with our proposal that the BaeA subunit may contribute more to catalysis by BaeAB than BaeB.

### BaeAB homologues from other sphingomonads stereospecifically cleave β(R)-MPHPV

Among the bacteria predicted to contain a homologue of BaeAB are *Novosphingobium* sp. PP1Y and *S. xenophagum*. The putative PP1Y-BaeA and PP1Y-BaeB polypeptides from *Novosphingobium* sp. PP1Y are 74 and 65% identical in amino acid sequence, respectively, to BaeA and BaeB from *N. aromaticivorans* (Table S1 and Fig. S7). The putative SxeBaeA and SxeBaeB polypeptides from *S. xenophagum* are 70 and 52% identical in amino acid sequence, respectively, to BaeA and BaeB (Table S1 and Fig. S7). To test whether BaeAB homologues from these species have the same activity as BaeAB from *N. aromaticivorans*, we cloned the predicted *baeA* and *baeB* genes from *Novosphingobium* sp. PP1Y and *S. xenophagum* into plasmids and expressed the recombinant proteins in *E. coli*. For both PP1Y-BaeAB and SxeBaeAB, two polypeptides were purified using Ni^2+^-chelate chromatography from the *E. coli* lysates, despite the fact that only the BaeA polypeptides contained a His_8_ tag (Fig. 3*B*, *lanes 5–8*), indicating that the proteins were heterodimers, like BaeAB from *N. aromaticivorans*. When we separately mixed PP1Y-BaeAB and SxeBaeAB with enantiopure samples of β(*S*)- or β(*R*)-MPHPV and GSH, neither enzyme cleaved the β(*S*)-MPHPV, but both enzymes converted β(*R*)-MPHPV into GS-HPV and guaiacol (Fig. 4 (*K–N*) and Fig. S8), precisely as we found with BaeAB from *N. aromaticivorans*.

## Discussion

Characterizing microbial strategies for lignin breakdown is important for understanding plant biomass turnover in nature and could aid in developing industrial systems for producing commodity chemicals from this abundant renewable resource. Here, we provide new information on the pathway used by sphingomonad bacteria to cleave the β-aryl ether bond commonly found in lignin. Specifically, we report on a previously uncharacterized heterodimeric β-etherase from *N. aromaticivorans* and other sphingomonads (BaeAB) with unique properties.

### BaeAB is a unique bacterial β-etherase

BaeAB is different from previously characterized bacterial β-etherases in several respects. Most notably, although BaeA and BaeB are phylogenetically distinct from the previously characterized β-etherases, they are more similar to the LigF homologues (which cleave the β(*S*)-aryl ether bond) than to the LigE homologues (which cleave the β(*R*)-aryl ether bond) (Fig. 7), even though BaeAB cleaves the β(*R*) isomer of the bond. This shows that the amino acid sequences of a β-etherase's polypeptides cannot alone be used to predict its stereospecificity.

In addition, BaeAB homologues are heterodimers, in contrast to the LigF and LigE homologues, which function as homodimers (25). Our conclusion that BaeAB is a heterodimer is supported by three lines of evidence. First, the individual BaeA and BaeB polypeptides showed little if any catalytic activity (Fig. 4, *B* and *C*), but activity was observed when they were combined or expressed in the same cell (Fig. 4, *D* and *J*). Second, both BaeA and BaeB were purified using Ni^2+^-chelate chromatography, despite the fact that only one polypeptide contained a His_8_ tag (Fig. 3*B*, *lane B3*). Finally, GPC analysis indicated that the purified BaeAB protein complex has an apparent molecular weight of ∼59 kDa (Fig. 5), whereas BaeA and BaeB are each predicted to have molecular weights of ∼29 kDa (Fig. 3).

The fact that substitution of a single amino acid residue (Asn^14^) in BaeA had a large negative effect on catalysis by BaeAB (Table 1), and our observation that amino acid sequence conservation appears to be more critical for BaeA than for BaeB (Fig. S7) suggests that the BaeA subunit makes a larger contribution to catalysis in BaeAB than in BaeB. One possible explanation for our results is that the main role of the BaeB subunit may be to form a dimer interface with BaeA that allows BaeA to correctly orient the β(*R*)-aryl ether substrate in its active site. Indeed, based on their three-dimensional structures, it was proposed that the homodimeric interfaces of LigE and LigF are important for determining their substrate stereospecificities (25). Another possible explanation for our result with the A:N14A variant enzyme is that the BaeB subunit has GST activity with some substrate(s) other than MPHPV.

### Diversity of β-etherases and implications for the evolution of the ability to break the β-aryl ether bond

Previous analyses of LigE and LigF homologues suggested that they are phylogenetically distinct from each other (29, 37). LigE from SYK-6 is ∼23% identical in amino acid sequence to members of the fungal FuA (formerly GTE, or etherase-like) GST class (23, 24), and its three-dimensional structure is most similar to those of members of this class (25). In contrast, structural analysis suggested that SYK-6 LigF may belong to a previously unrecognized class of GSTs (25). In a GST classification system based on a similarity network analysis, LigE is a member of the Main.28 subgroup, and LigF is not a member of any established Main subgroup (38). Our analysis shows that LigF homologues are phylogenetically more similar to GST_Nu_ homologues (members of the Nu class of GSTs (26)) and LigG homologues (which share some features of the Omega class (27)) than to the LigE homologues (Fig. 7). In addition, our analysis suggests that there are two related, yet distinct, LigF homologue subclades, which we refer to as the LigF1 and LigF2 homologues (Fig. 7).

It is unclear how best to classify BaeA and BaeB. Neither are members of an established Main subgroup in the similarity network GST classification system (38). Despite being phylogenetically related to the LigF homologues, BaeA and BaeB may not warrant formal inclusion in the LigF GST class, because of their low amino acid sequence homologies to other members of this class (<36% and <30% identical, respectively; Fig. S6) (an amino acid sequence identity of ∼40% is often used as a cutoff for inclusion in a GST class (37)). The low sequence identity between BaeA and BaeB (∼24%; Fig. S6) also argues against them being members of the same GST class as each other, although, paradoxically, the fact that they hybridize together argues for classifying them as members of the same class, as GST heterodimers are typically composed of members of the same class, and the ability to hybridize is another criterion used in GST classification (31).

Because the LigE and LigF homologues are so different in structure and amino acid sequence, it has been proposed that they evolved independently to specifically cleave either the β(*R*) or β(*S*) stereoisomer of the β-aryl ether bond, respectively (25). It was recently reported that the FuA GST, Ds-GST1, from the white-rot fungus *Dichomitus squalens* specifically cleaves the β(*S*)-aryl ether bond (39), although it clusters closer to the LigE homologues than to the LigF homologues in our phylogenetic tree (Fig. 7). Together with the unexpected stereospecificity of BaeAB, these results suggest that the ability to break the β-aryl ether bond did arise separately in the LigF/BaeAB and FuA/LigE GST clusters but that, even though every individual β-etherase characterized thus far has been stereospecific, neither cluster evolved to specifically cleave only one of the stereoisomers of the bond. In addition, not all members of these two clusters appear to be capable of breaking the β-aryl ether bond, as demonstrated by the inability of LigE-like GSTs from *Sorangium cellulosum* (28) and *Rhodopseudomonas palustris* (29) (which share ∼39 and ∼37% sequence identity with NaLigE, respectively) to cleave substrates containing either stereoisomer of the bond (Fig. 7).

Our finding that BaeA Asn^14^ is involved in catalysis by BaeAB suggests that the analogous asparagine present in the LigF homologues (Fig. 6*A*) may also be involved in catalysis by those enzymes. The proximity of the side-chain amide nitrogen of Asn^13^ to the GSH thiol in the LigF structure (3.3 Å; Fig. 6*B*), along with the fact that Ser^14^ is the only other residue with a polar side chain near the thiol in the LigF structure (Fig. 6*B*), suggest that the conserved asparagine may be involved in activating the GSH cofactor (which is typically stabilized in its thiolate form via hydrogen-bonding with a residue side chain in characterized GSTs (34)). If the conserved asparagine were activating the GSH in these enzymes, they would be unique among characterized GSTs, which typically use a residue with a side-chain hydroxyl (such as tyrosine) to activate the GSH (31). It would also suggest different reaction mechanisms between the LigF/BaeAB and FuA/LigE β-etherases, as LigE does not contain an active-site asparagine (25) and Ds-GST1 is not predicted to contain one (39). Interestingly, although NaLigE and BaeAB apparently evolved the ability to cleave the β(*R*)-aryl ether bond separately, and presumably utilize different reaction mechanisms, the catalytic efficiencies of these two enzymes are comparable (Table 1).

### The relative roles of BaeAB and LigE in breaking the β(R)-aryl ether bond

The fact that *N. aromaticivorans* strains deficient in either LigE (12444ΔligE) or BaeAB (12444Δ2872) metabolized MPHPV more slowly than their parent strain (12444Δ1879) (Fig. 2, *A–C*) shows that both enzymes contribute to metabolism of β(*R*)-MPHPV in WT *N. aromaticivorans*, although neither individual enzyme is essential. In addition, the fact that strains lacking both BaeAB and NaLigE (12444ΔligEΔ2872 and 12444ΔligEΔ2873) were unable to fully metabolize MPHPV (Fig. 2, *D* and *E*) suggests that these are the only enzymes that *N. aromaticivorans* uses to cleave β(*R*)-MPHPV. These results suggest that individual organisms may only require either a LigE or BaeAB homologue to metabolize compounds containing the β(*R*)-aryl ether bond. Indeed, the genome of *Sphingobium* sp. SYK-6, which encodes two active LigE homologues (LigE and LigP) (11), does not contain a putative BaeAB homologue (Table S1). Conversely, *Sphingobium* sp. YG1, which is reported to metabolize GGE (40), contains genes for a putative BaeAB homologue but is not predicted to encode a LigE homologue (Table S1). The fact that an *N. aromaticivorans* strain containing both BaeAB and NaLigE metabolized MPHPV faster than mutants lacking one of these enzymes (Fig. 2, *A–C*) also suggests that the reason that some organisms contain both a BaeAB and a LigE homologue (or two LigE homologues) (Table S1) may be that it increases the intracellular rate of β-aryl ether bond breaking. Otherwise, although BaeAB and NaLigE reacted similarly with β(*R*)-MPHPV (Table 1), the enzymes may react differently from each other with other oligomers containing the β(*R*)-aryl ether bond, as would be derived from real lignin.

### Conclusions

Prior to this work, LigE homologues were the only known bacterial enzymes capable of specifically breaking the β(*R*)-aryl ether bond in a defined way. We showed that BaeAB, which is distinct from previously characterized β-etherases in several ways, can perform this reaction at least as well as LigE homologues and that BaeAB homologues may be as widespread among bacteria as LigE homologues. These results suggest that the previously unrecognized BaeAB homologues may play an important role in degradation of lignin-derived aromatic oligomers by sphingomonad bacteria in nature. In sum, our work shows that there is more variability among individual sphingomonads in their pathway for breaking the β-aryl ether bond than previously thought and illustrates the importance of studying this pathway, and probably other pathways involved in metabolizing lignin-derived compounds, in multiple species in order to better understand this diversity.

## Experimental procedures

### N. aromaticivorans strains

*N. aromaticivorans* 12444Δ1879 is a derivative of WT strain DSM 12444 (also called F199 (41, 42)), in which Saro_1879 (putative *sacB*; also called SARO_RS09410) has been deleted from the genome to create a strain amenable to markerless genomic modifications using a *sacB*-containing plasmid (26). We used 12444Δ1879 as parent strain to generate strains 12444ΔligE (lacking Saro_2405 (*ligE*; also called SARO_RS12100)), 12444Δ2872 (lacking Saro_2872 (also called SARO_RS14565)), 12444ΔligEΔ2872 (lacking both Saro_2405 and Saro_2872), and 12444ΔligEΔ2873 (lacking both Saro_2405 and Saro_2873 (also called SARO_RS14570)). Methods for constructing mutants are contained in the supporting information, and primers used are contained in Table S2. Fig. S2 shows genotypes for all deletion mutants.

### Bacterial growth media

*E. coli* strains used for creating plasmids were grown in lysogeny broth, and shaken at ∼200 rpm at 37 °C. For routine storage and manipulation, *N. aromaticivorans* cultures were grown in lysogeny broth or GluSis at 30 °C. GluSis is a modification of Sistrom's minimal medium (43) in which the succinate has been replaced by 22.6 mm glucose. *N. aromaticivorans* growth experiments used standard mineral base (SMB) minimal medium (26), at an initial pH of 7.0. Where needed to select for the presence or absence of plasmids, media were supplemented with 100 μg/ml ampicillin, 50 μg/ml kanamycin, 20 μg/ml chloramphenicol, or 10% sucrose (w/v).

### N. aromaticivorans growth experiments

Starter cultures of *N. aromaticivorans* were grown in 4 ml of SMB containing 4 mm vanillate. Experimental cultures were grown in 20–30 ml of SMB containing 3 mm vanillate and 1 mm *erythro*-GGE, in 125-ml conical growth flasks shaken at 200 rpm at 30 °C. Aliquots (400–600 μl) were removed at various time points and passed through 0.22-μm filters (*e.g.* Whatman Puradisc filters; GE Healthcare) before HPLC analysis of extracellular aromatics. Every culture was grown at least three times; data shown are from representative cultures.

For the 12444ΔligEΔ2872 and 12444ΔligEΔ2873 cultures, we filtered >2 ml for the final time points. To determine which stereoisomer(s) of MPHPV were present in these samples, they were split into three 400-μl aliquots and combined with 5 mm GSH and either H_2_O, recombinant NaLigE (90 μg/ml), or recombinant NaLigF1 (147 μg/ml) and incubated at 30 °C for 1 h. These samples were analyzed via HPLC to assay for MPHPV cleavage into GS-HPV and guaiacol as described below.

### Expression and purification of recombinant enzymes

We separately cloned Saro_2873 (encoding BaeA (WP_011446513.1)), Saro_2872 (encoding BaeB (WP_011446512.1)), or Saro_2405 (encoding NaLigE (WP_011446047.1)) from *N. aromaticivorans* DSM 12444 into plasmid pVP302K (29) so that each translated polypeptide would contain a His_8_ tag connected to the N terminus, with an intervening peptide containing a TEV protease recognition site. We also separately cloned both Saro_2873 and Saro_2872, both PP1Y_AT11532 and PP1Y_AT11540 from *Novosphingobium* sp. PP1Y (encoding PP1Y-BaeA (WP_013832467.1) and PP1Y-BaeB (WP_013832468.1), respectively), or both SX1_RS06450 and SX1_RS06455 from *S. xenophagum* NBRC 107872 (encoding SxeBaeA (WP_019052344.1) and SxeBaeB (WP_019052345.1), respectively) into pVP302K. In each plasmid containing two genes, a His_8_ tag with intervening TEV protease recognition site was included upstream of only one of the genes (so only the BaeA or BaeB polypeptide would contain the His_8_ tag on its N terminus). Plasmids containing both Saro_2873 and Saro_2872 were modified via PCR to generate plasmids for expressing BaeAB variants containing alanine substitutions for Ser^15^ in BaeA (A:S15A), Ser^14^ in BaeB (B:S14A), both BaeA Ser^15^ and BaeB Ser^14^ (A:S15A/B:S14A), and Asn^14^ in BaeA (A:N14A). Construction of plasmids is described in the supporting information.

Expression plasmids were transformed into *E. coli* B834 (44, 45) containing plasmid pRARE2 (Novagen). Recombinant proteins were expressed by growing the *E. coli* strains for ∼25 h at 25 °C in ZYM-5052 autoinduction medium (46) containing kanamycin and chloramphenicol. Recombinant proteins were purified by passing crude *E. coli* lysates through a column packed with Ni^2+^-NTA resin (Qiagen or Thermo Scientific) as described previously (26), except using gravity-flow columns instead of an FPLC system. After incubation of purified proteins with His_6_-TEV protease, the polypeptides that originally contained a His_8_ tag retained a Ser-Ala-Ile-Ala-Gly- peptide on their N termini, derived from the linker between the protein and the TEV protease recognition site. (For BaeAB with the His tag on BaeB, Met^1^ was not coded for, so the SAIAG- peptide was linked to residue Ser^2^.) The cleaved His_8_ tags and His_6_-TEV protease were removed via a second round of Ni^2+^-NTA resin chromatography. Recombinant NaLigF1 and a version of NaLigE that had originally contained a His tag on its C terminus were purified as described previously (29). Recombinant protein concentrations were determined via the Bradford method (absorbance at 595 nm), using BSA standards of known concentrations (Pierce, Thermo Scientific) and protein assay dye reagent from Bio-Rad.

### Cell-free protein synthesis

We individually cloned Saro_2872 and Saro_2873 into plasmid pEU-NGFP (47), removing the GFP gene in the process (for details, see supporting information). Cell-free protein synthesis was performed essentially as described previously (48). The Saro_2872 polypeptide (BaeB) contained a His_6_ tag with an intervening TEV protease recognition site on its N terminus that was not removed. The Saro_2873 polypeptide (BaeA) was synthesized in its native form. Synthesized polypeptides were assayed for enzyme activity using aliquots directly from the cell-free synthesis reaction. Concentrations of BaeA and BaeB in the synthesis reaction mixtures were approximated using the intensities of the polypeptides in an SDS-polyacrylamide gel (49).

### Gel permeation chromatography

A Wyatt (Santa Barbara, CA) size-exclusion chromatography analytical column for membrane proteins (5 μm, 300 Å, 7.8 × 300 mm) was attached to a Prominence HPLC (Shimadzu; Kyoto, Japan) equipped with an LC-20AD pump, a CTO-20A column oven (35 °C), and a SPD-M20A diode array detector (monitoring 250–500 nm). The buffer was 5 mm HEPES (pH 7.5), 100 mm NaCl, and 0.5 mm tris(2-carboxyethyl)phosphine, run at a flow rate of 1 ml/min. Protein retention times were determined using absorbance at 280 nm. Proteins of known molecular weights used to generate a standard curve were purchased from Sigma-Aldrich (bovine heart cytochrome *c*, *Glycine max* trypsin inhibitor, bovine erythrocyte carbonic anhydrase, and BSA) or GE Healthcare (RNase A, ovalbumin, and conalbumin; contained in a gel filtration calibration kit), except for NaGST_Nu_, which was purified as described previously (26).

### Enzyme activity assays

For assays with cell-free synthesized BaeA and BaeB polypeptides, 0.1 mm racemic (β(*S*) and β(*R*)) MPHPV was combined with 5.8 mm GSH in enzyme reaction buffer (ERB; 25 mm Tris-HCl (pH 8.0) and 25 mm NaCl). Cell-free protein synthesis mixtures containing BaeA or BaeB were added individually or together to the MPHPV/GSH solutions to achieve concentrations of ∼24 nm of each polypeptide. These 625-μl reactions were incubated at 30 °C for 24 h to several days. Each was then split into 190-μl aliquots and combined with an additional 2.3 mm GSH and either H_2_O, 151 μg/ml NaLigE, or 184 μg/ml NaLigF1. These 212-μl reactions were incubated at 30 °C for several hours and then analyzed via HPLC.

For assays with recombinant BaeAB, PP1Y-BaeAB, or SxeBaeAB, 0.1–0.6 mm racemic (β(*S*) and β(*R*)) MPHPV was first combined with 5 mm GSH in ERB containing 0–1.5% DMSO (to increase the solubility of MPHPV; no effect of DMSO on enzyme activity was observed). Recombinant NaLigE (144 μg/ml) or NaLigF1 (236 μg/ml) was added to separate 1.1-ml aliquots of the racemic MPHPV mixture and allowed to react at 30 °C for 1.5 h to generate enantiopure samples of β(*S*)-MPHPV or β(*R*)-MPHPV, respectively. Aliquots of these enantiopure samples were combined with H_2_O, 77 μg/ml BaeAB, 98 μg/ml PP1Y-BaeAB, or 91 μg/ml SxeBaeAB. These reactions were incubated at 30 °C for several hours and then analyzed via HPLC.

### Kinetics of the enzymatic cleavage of β(R)-MPHPV

Various concentrations of racemic (β(*S*) and β(*R*)) MPHPV were combined with 5 mm GSH in ERB. At time 0, 100 μl of an indicated enzyme in ERB + 5 mm GSH was combined with 1 ml of the racemic MPHPV/GSH sample (both pre-equilibrated to 25 °C) at 25 °C. The final concentration of β(*R*)-MPHPV in each reaction was 0.0045, 0.010, 0.017, 0.068, or 0.13 mm. Final enzyme concentrations were 18 nm BaeAB, 23 nm BaeAB (B:S14A), 22 nm BaeAB (A:S15A), 24 nm BaeAB (A:S15A/B:S14A), 98 nm BaeAB (A:N14A), and 70 nm NaLigE (all BaeAB concentrations are for the heterodimers; the NaLigE concentration is for the individual polypeptides that make up the functional homodimers). At different time points, 200 μl of a reaction was removed and combined with 40 μl of 1 m HCl (Acros Organics, Geel, Belgium) to terminate the enzyme reaction before HPLC analysis to quantify GS-HPV formed. Control reactions were allowed to proceed for several hours to ensure that only the β(*R*)-MPHPV in the reaction mixtures was cleaved.

### HPLC analysis

Analysis and quantification of aromatic compounds were performed using an Ultra AQ C18 (5-μm; 250 × 4.6-mm) column (Restek, Bellefonte, PA) attached to a System Gold HPLC (Beckman Coulter, Brea, CA) with buffers described in Fig. S9. In this system, the following pairs of stereoisomers ran as single peaks: α(*S*)β(*R*) and α(*R*)β(*S*) *erythro*-GGE, α(*R*)β(*R*) and α(*S*)β(*S*) *threo*-GGE, β(*R*) and β(*S*) MPHPV, and β(*R*) and β(*S*) GS-HPV. The eluent was analyzed for light absorbance between 191 and 600 nm, and absorbances at 280 nm were used for quantification of aromatic metabolites by comparing peak areas with those of standards.

### Chemicals

Vanillate, guaiacol, and GSH were purchased from Sigma-Aldrich. *Erythro*-GGE was purchased from TCI America (Portland, OR). Racemic MPHPV and HPV were synthesized as described previously (26).

### Phylogenetic analysis of GSTs involved in breaking the β-aryl ether bond

Proteins used in the analysis are listed in Table S3. Proteins were chosen by performing BLASTp searches of the NCBI nonredundant protein database using the amino acid sequences of NaLigE, NaLigF1, NaLigF2, BaeA, and BaeB as queries. Every third hit up to 25 proteins total from each search was combined into a single list, and any duplicate hits from the different searches were removed. To this list were added all of the GSTs that have been tested for the ability to perform one of the reactions involved in the sphingomonad pathway for breaking the β-aryl ether bond, if they were not already part of the list (see legend to Fig. 7). Protein sequences were aligned using MAFFT in MegAlign Pro, which is part of the Lasergene version 14.0 suite (DNASTAR, Madison, WI). A phylogenetic tree was calculated via the maximum likelihood method in RAxML version 8.2.3 (50), using 100 rapid bootstrap inferences. The tree was visualized using Interactive Tree of Life version 3 (51).

## Author contributions

W. S. K. conceptualization; W. S. K. and C. N. O. data curation; W. S. K., C. N. O., and L. M. Y. formal analysis; W. S. K., C. N. O., L. M. Y., A. V. N., and K. A. W. investigation; W. S. K., C. N. O., L. M. Y., E. T. B., K. A. V. M., S. D. K., and D. L. G. methodology; W. S. K. writing-original draft; W. S. K., E. T. B., and T. J. D. writing-review and editing; D. R. N. and T. J. D. resources; D. R. N. and T. J. D. funding acquisition; T. J. D. supervision.

## Supplementary Material

Supporting Information

## Footnotes

3 W. S. Kontur, unpublished observation.

## Footnotes

2 The abbreviations used are:

GGE: guaiacylglycerol-β-guaiacyl ether
GS: conjugated glutathione moiety
GST: glutathione *S*-transferase
MPHPV: β-(2-methoxyphenoxy)-γ-hydroxypropiovanillone
GS-HPV: β-glutathionyl-γ-hydroxypropiovanillone
HPV: hydroxypropiovanillone
TEV: tobacco etch virus
SMB: standard mineral base
GPC: gel permeation chromatography
ERB: enzyme reaction buffer
NTA: nitrilotriacetic acid
BisTris: 2-[bis(2-hydroxyethyl)amino]-2-(hydroxymethyl)propane-1,3-diol.
