# Supplementary material for: A heterodimeric glutathione S-transferase that stereospecifically breaks lignin's β(R)-aryl ether bond reveals the diversity of bacterial β-etherases
Source: J Biol Chem. 2018 Dec 12;294(6):1877–90. doi: 10.1074/jbc.RA118.006548 (PMC6369299; doi:10.1074/jbc.RA118.006548)
Supplement: Supporting Information [file supp_294_6_1877__index.html]

A heterodimeric glutathione S-transferase that stereospecifically breaks lignin’s β(R)-aryl ether bond reveals the diversity of bacterial β-etherases — Discovery of a heterodimeric bacterial β-etherase — A heterodimeric glutathione S-transferase that stereospecifically breaks lignin's β(R)-aryl ether bond reveals the diversity of bacterial β-etherases — Discovery of a heterodimeric bacterial β-etherase — Supporting Information 

# A heterodimeric glutathione *S*-transferase that stereospecifically breaks lignin's β(*R*)-aryl ether bond reveals the diversity of bacterial β-etherases

## Supporting Information

- Phylogenetic tree (Fig. 7) newick file - This file contains the coordinates needed to recreate the phylogenetic tree in Fig. 7. It is a newick file saved as a text file (an on-line program like Interactive Tree of Life can read this file to re-create the Fig. 7 tree)
- Supporting Information - Detailed methods and supplemental figures and tables
