# Supplementary material for: A heterodimeric glutathione S-transferase that stereospecifically breaks lignin's β(R)-aryl ether bond reveals the diversity of bacterial β-etherases
Source: J Biol Chem. 2018 Dec 12;294(6):1877–90. doi: 10.1074/jbc.RA118.006548 (PMC6369299; doi:10.1074/jbc.RA118.006548)
Supplement: Supporting Information [file supp_RA118.006548_141721_1_supp_244154_pjclch.pdf]

SUPPORTING INFORMATION

A heterodimeric glutathione S-transferase that stereospecifically breaks lignin's β(*R*)-aryl ether bond reveals the diversity of bacterial β-etherases

Wayne S. Kontur[1,2], Charles N. Olmsted[1,2], Larissa M. Yusko[1,2], Alyssa V. Niles[1,2], Kevin A. Walters[1,2], Emily T. Beebe[1,2,3], Kirk A. Vander Meulen[1,2,3], Steven D. Karlen[1,2,3], Daniel L. Gall[1,2], Daniel R. Noguera[1,2,4], and Timothy J. Donohue[1,2,5,*]

From the [1]Wisconsin Energy Institute, the [2]Department of Energy Great Lakes Bioenergy Research Center, and the Departments of [3]Biochemistry, [4]Civil and Environmental Engineering, and [5]Bacteriology, University of Wisconsin, Madison, Wisconsin 53706

[*]To whom correspondence should be addressed: Dept. of Bacteriology, University of Wisconsin, Madison, WI 53706. Tel.: 608-262-4663; E-mail: tdonohue@bact.wisc.edu.

<u>Table of Contents</u>

Construction of *Novosphingobium aromaticivorans* mutants……………………..……………page S-2
Expression of recombinant proteins in *E. coli*……………………………………..……….…page S-3
Cell-free synthesis of BaeB (Saro_2872) and BaeA (Saro_2873)……………………………page S-5
Figure S1...…………………………………………………………………………………page S-6
Figure S2...…………………………………………………………………………………page S-7
Figure S3...…………………………………………………………………………………page S-8
Figure S4...…………………………………………………………………………………page S-9
Figure S5...…………………………………………………………………………………page S-10
Figure S6...…………………………………………………………………………………page S-11
Figure S7...…………………………………………………………………………………page S-12
Figure S8...…………………………………………………………………………………page S-13
Figure S9...…………………………………………………………………………………page S-14
Table S1....…………………………………………………………………………………page S-15
Table S2....…………………………………………………………………………………page S-19
Table S3....…………………………………………………………………………………page S-21
References....……………………………………………………………………………page S-24

A Newick file (in text format) for the phylogenetic tree in Fig. 7 in the main text is included as a separate file

<u>Construction of *Novosphingobium aromaticivorans* mutants</u>

Biological reagents

All PCR reactions were performed with Herculase II polymerase (Agilent Technologies, Santa Clara, CA). Primers were phosphorylated with polynucleotide kinase from Promega (Madison, WI). All other enzymes were from New England Biolabs (Ipswich, MA). All primers were from Integrated DNA Technologies (Coralville, IA). Primers are listed in Table S2. For cloning using the NEBuilder HiFi Assembly system (New England Biolabs), plasmid pK18msB-MCS was linearized via PCR as previously described (1).

Plasmid for deleting *ligE* (Saro_2405)

A region of the *N. aromaticivorans* genome extending from 1501 bp upstream of Saro_2405 to 1503 bp downstream of the gene was amplified from genomic DNA using primers "pK18-ligE OvExt F" and "pK18-ligE OvExt R", which contain 5' ends that are complementary to the ends of linearized pK18msB-MCS. The genomic DNA fragment was combined with linearized pK18msB-MCS using the NEBuilder HiFi Assembly system to produce plasmid pK18msB-ligE. This plasmid was amplified using kinase phosphorylated primers "Saro2405 ligE del F" and "Saro2405 ligE del R" to produce a linear fragment in which the majority of Saro_2405 (including the start codon and a short region upstream of the start codon) was missing (Fig. S2). This linear fragment was circularized using T4 DNA Ligase to generate plasmid pK18msB-ΔligE.

Plasmid for deleting *baeB* (Saro_2872)

A region of the *N. aromaticivorans* genome extending from 1073 bp upstream of Saro_2872 to 954 bp downstream of the gene was amplified from genomic DNA using primers "Saro_2872 ampl AseI F2" and "Saro_2872 ampl XbaI R", which contain recognition sites for the restriction enzymes AseI or XbaI, respectively, incorporated into their 5' ends. The resulting fragment was digested with AseI and XbaI, then ligated with pK18msB-MCS (1) that had been digested with AseI and XbaI, using T4 DNA Ligase, to form plasmid pK18msB-Saro2872. This plasmid was amplified using kinase phosphorylated primers "Saro_2872 del R" and "Saro_2872 del F" to produce a linear fragment in which the majority of Saro_2872 was missing (Fig. S2). Since the start codon of Saro_2872 overlaps with the stop codon of Saro_2873, "Saro_2872 del F" contains a single base mismatch with pK18msB-Saro2872 (Table S2), to inactivate the Saro_2872 start codon, while preserving the Saro_2873 stop codon. This linear fragment was circularized using T4 DNA Ligase to generate plasmid pK18msB-ΔSaro2872.

Plasmid for deleting *baeA* (Saro_2873)

Approximately 1100 bp regions from upstream and downstream of Saro_2873 were separately amplified from genomic DNA using primer sets "Saro_2873-pk18 hifi ampl R" and "Saro_2873 hifi del F", and "Saro_2873-pk18 hifi ampl F" and "Saro_2873 hifi del R", respectively. These two fragments were combined with linearized pK18msB-MCS using the NEBuilder HiFi Assembly system to produce plasmid pK18msB-ΔSaro2873, in which the regions that naturally flank Saro_2873 in the genome are adjacent to each other (Fig. S2).

Deleting genes from the *N. aromaticivorans* genome

Plasmids for deleting individual genes were separately mobilized into *N. aromaticivorans* via conjugation with *Escherichia coli* S17-1. For the conjugation, cultures of *E. coli* S17-1 harboring the plasmid (in LB containing kanamycin) and *N. aromaticivorans* (in GluSis) were grown overnight at 30 °C. Cultures were diluted and allowed to resume exponential growth before being harvested by centrifugation. Cell pellets were washed in LB, then resuspended together into 90 µL LB. Conjugations were allowed to proceed overnight at 30 °C. The following day, the cells were harvested via centrifugation, resuspended into GluSis, and shaken at 200 rpm for >1 h at 30 °C. Outgrown cells were then plated onto solid GluSis with kanamycin to select for *N. aromaticivorans* cells in which the plasmid had incorporated into the genome via homologous recombination (single crossovers). Single crossovers were confirmed through the inability to immediately grow on GluSis containing 10% sucrose.

Single crossovers were cultured in 5 mL of GluSis containing 10% sucrose and shaken at 30 °C until growth commenced (usually after several days), which signified loss of the plasmid from the genome via a second

round of homologous recombination. These cultures were streaked onto solid GluSis containing 10% sucrose to isolate individual strains that has lost the plasmid, and plasmid loss was confirmed by the inability to grow on GluSis containing kanamycin. The loss of each desired gene was confirmed via PCR and sequencing of isolated genomic DNA.

Expression of recombinant proteins in *E. coli*

Plasmids for expressing recombinant BaeB (Saro_2872)

Saro_2872 was amplified from *N. aromaticivorans* genomic DNA with the primers "Saro2872 Ctag BsaI F" and "Saro2872 Ctag BspHI R". This fragment was digested with restriction enzymes BspHI and BsaI. The expression vector pVP302K (2) was amplified using primers "pVP302K Ctag BsaI F" and "pVP302K Ctag NcoI R", and the resulting fragment was digested with BsaI and NcoI. The digested fragments were combined using T4 DNA ligase, generating plasmid pVP302K/Ctag-2872, which consists of a T5 promoter followed by the coding sequences of Saro_2872 (lacking its stop codon), the RtxA protease from *Vibrio cholerae*, and a His$_8$ tag.

pVP302K/Ctag-2872 was amplified using kinase phosphorylated primers "Ctag 2872-pVP add Stop R" and "pVP302K Ntag HindIII F". This fragment was circularized using T4 DNA ligase to generate plasmid pVP302K/Untagged2872, in which a stop codon (TAA; Table S2) has been introduced directly after Saro_2872.

pVP302K/Untagged2872 was amplified via PCR using kinase phosphorylated primers "2872-pVP C to Ntag F" and "pVP302 C to Ntag R". The amplified fragment was circularized using T4 DNA ligase to generate plasmid pVP302K/Ntag-2872, which contains a T5 promoter followed by coding sequences for a His$_8$-tag, a tobacco etch virus (TEV) protease recognition site and Saro_2872.

Plasmids for expressing recombinant BaeAB (Saro_2872 and Saro_2873)

To express BaeAB containing a His$_8$-tag on the N-terminus of BaeB (Saro_2872):

We initially generated a strain of *N. aromaticivorans* in which a coding sequence for a His$_8$-tag was incorporated into the genome so that the Saro_2872 protein would contain a His$_8$-tag on its N-terminus. Plasmid pK18msB-Saro2872 was amplified via PCR using kinase phosphorylated primers "Saro2872 gNtag R" and "Saro2872 gNtag F", to generate a fragment containing Saro_2873 (with its stop codon), followed by a coding sequence for a His$_8$-tag, then a TEV protease recognition site, then Saro_2872 (missing its native start codon). This fragment was circularized using T4 DNA ligase to generate plasmid pK18msB-H$_8$Saro2872. pK18msB-H$_8$Saro2872 was mobilized into strain 12444Δ2872 via conjugation from *E. coli* S17-1, and a strain of *N. aromaticivorans* (12444-H$_8$2872) containing the coding sequence for Saro_2872 containing an N-terminal His$_8$-tag was generated and isolated using homologous recombination as described above for generating deletion mutants.

We performed PCR with genomic DNA from strain 12444-H$_8$2872 as template using primers "2872-3_pVP_HiFi_F" and "Saro2872-3NOTAG_pVP_HiFi_R" to generate a fragment containing the coding sequence for Saro_2873 (with native stop codon intact), followed by the coding sequence for a His$_8$-tag, then for a TEV protease recognition site, then Saro_2872 (lacking its start codon), with extensions on the ends of the fragment that are complementary to plasmid pVP302K. pVP302K was amplified via PCR using the primers "pVP302K-HiFi-noTag-R" and "pVP302K-HiFi-ATW-F". These two fragments were combined using the NEBuilder HiFi Assembly system to create plasmid pVP302K/2873-H2872.

The amino acid sequence of His$_8$-tagged BaeB is (with TEV protease recognition site underlined, and Ser^2 of the native protein in bold; note that Met^1 of the native protein was not coded for):

M I H H H H H H H H A L A S E N L Y F Q S A I A G **S** A L L Y H G E P N G A S L T V L A A L A E T G L
D I E C R R I D L L A G E R H S L P G I V D P V A L D L S I E G E G P V L V I D G E A M T E S V F L A Q
Y L D E A A G G V G L Q P T D A Y A R W E M M M W C R Q I T E R L S P A A A L L G N L A T S Q S A
I A A I P A E D F A I L A A R I V S D D L R E R W Q A L N D D A V N A A Q V A D S E T K V A A A V D
R C E K Q L G D G R E W L M G T F S I A D L V T Y S W L A G M E P L R P A A F A D A P L V K A W L
A R T A A R P C V Q A A L A R A T I S E P L R A W A P G P E I N R W G

To express BaeAB containing a His$_8$-tag on the N-terminus of BaeA (Saro_2873):

We performed PCR with genomic DNA from strain 12444Δ1879 as template using primers "2872-3_pVP_HiFi_F" and "Saro2872-3Ntag_pVP_HiFi_R" to generate a fragment containing the native genomic organization of the Saro_2873 and Saro_2872 genes, with regions on the ends of the fragment that are complementary to plasmid pVP302K. pVP302K was amplified via PCR using the primers "pVP302K-HiFi-ATW-R" and "pVP302K-HiFi-ATW-F". These two fragments were combined using the NEBuilder HiFi Assembly system to create plasmid pVP302K/H2873-2872.

<u>To express BaeAB amino acid substitution mutants</u>
To generate mutant B:S14A, plasmid pVP302K/2873-H2872 was amplified by kinase phosphorylated primers "Saro2872-S14A_R" and "Saro2872-S14A_F". To generate mutant A:S15A, plasmid pVP302K/H2873-2872 was amplified by kinase phosphorylated primers "Saro2873-S15A_R" and "Saro2873-S15A_F". To generate mutant A:N14A, plasmid pVP302K/2873-H2872 was amplified by kinase phosphorylated primers "Saro2873-S15A_R" and "Saro2873-N14A_F". These linear fragments were separately circularized using T4 DNA ligase to generate plasmids pVP302K/2873-H2872(S14A), pVP302K/H2873(S15A)-2872, and pVP302K/2873(N14A)-H2872, respectively.

To generate mutant A:S15A/B:14A, plasmid pVP302K/2873-H2872(S14A) was amplified by kinase phosphorylated primers "Saro2873-S15A_R" and "Saro2873-S15A_F". The linear fragment was circularized using T4 DNA ligase to generate plasmid pVP302K/2873(S15A)-H2872(S14A).

Plasmids for expressing recombinant BaeA (Saro_2873)
Plasmids pVP302K/2873-H2872 and pVP302K/H2873-2872 were amplified via PCR using kinase phosphorylated primers "pVP302K-HiFi-ATW-F" and "Saro_2872 del F". These fragments were separately circularized using T4 DNA ligase to generate plasmids pVP302K/Untagged2873 and pVP302K/Ntag-2873, respectively.

Plasmids for expressing recombinant *Novosphingobium* sp. PP1Y and *Sphingobium xenophagum* BaeAB homologues
Regions containing the overlapping *baeAB* genes were amplified from *Novosphingobium* sp. PP1Y and *Sphingobium xenophagum* NBRC 107872 genomic DNA using primer sets "PP1Y_BaeE_Ntag_pVP_HiFi_F" and "PP1Y_BaeE_Ntag_pVP_HiFi_R", and "Sxeno-BaeE_Ntag_pVP_HiFi_F" and "Sxeno-BaeE_Ntag_pVP_HiFi_R", respectively. The resulting fragments contained regions at their ends that were complimentary to plasmid pVP302K. pVP302K was amplified via PCR using the primers "pVP302K-HiFi-ATW-R" and "pVP302K-HiFi-ATW-F". The two amplified genomic fragments were combined with the linear pVP302K fragment using the NEBuilder HiFi Assembly system to create plasmids pVP302K/PP1Y-baeAB and pVP302K/Sxeno-baeAB.

The amino acid sequence of His_8-tagged PP1Y-BaeA is (with TEV protease recognition site underlined, and Met^1 of the native protein in bold):
M G H H H H H H H H A L A S <u>E N L Y F Q S</u> A I A G **M** A Q V T L Y H W E P N A N S G K P M L T L M
E K G V E F D S H Y I D M L E F D Q H R P E Y L A I N P Q G T I P A M T H G S R V L V E S T A I M E Y
V N E E F S G P D L M P K D A L D R W R V R W W M K F M D Q W L A P S F S M I G W S V F V G P M
V R Q R D P A E L E A A I E R I P L P E R R V S W R K A I H G T F S E A E I G E S Q R R V A L G I G M L
E Q E L G K R E W L A S D A Y S L A D I N G F N L A Y A I P L S Q P S L C N D E R T P N L L R W L R
A V Y A R P A V K K C W A L G R T D M V K R V A I L D G E Q I

The amino acid sequence of native PP1Y-BaeB is:
M A M I L Y H G A P N G P S L I V L A A L A E T G V A V E R R R I D L L A G D R H V L P G I S E P V A
L N M G I E G E G P V L V V D G E A M T D A V F L A Q Y F D E L S E K A S L Q P S D P Y A H W E M
M M W C R Q I T E R L S P A A A L L G N V E F S Q E T L G A M S D E Q F E E L T D R I V S A D L R S
R W E I L R D G A V D L A Q I T D S R T K V L Q A V E R C E Q Q L G D G R E W L M G E L T I A D L T
T F A W L A G M E Q V L P E S F R D K P L T T A W L Q R V R S R P A V A S A L A D G G A R A A T F
W A P G P E I N R W G

The amino acid sequence of His_8-tagged Sxe-BaeA is (with TEV protease recognition site underlined, and Met[1] of the native protein in bold):
M G H H H H H H H H A L A S E N L Y F Q S A I A G **M** T D V T L Y H W E P N A N S G K P M L A L F E
K G V A F D S H Y L D L L N F D Q H K P D Y L A V N P L G T I P A M T H G Q H V L T E S T A I M E Y
V D E A F D G P R L M P V D P V D Q W R T R W W M K F L D Q W L A P S F S M I G W S V F V G P S V
R Q K D P A E L E A A I D R I P M P E R R I A W R K A I N G A F S A E E M A E S Q R R V A L G I T Y L
E Q A L G Q R D W L A S N S Y G L A D I N G F N L A Y A M P L S Q P Q L C N D D L T P N I M R W L
R A I Y A R P A T R A C W A L G R T D L A R R I S L L E S E P A

The amino acid sequence of native Sxe-BaeB is:
M T R I L Y H G Q P N G P S F T V L A A A F E K D V T L D L R E F D L V A G D R H A P A L P H P I E V
D Q S I E G E G P V F I V D G V A M T D S V F L A C Y L D E I G S G P A L R P A D P Y A R W Q M M A
W C R Y V I E R V A P A A A C L G V A A H P P A A V P A G I A S A D L E Q R W R D A V E G R A D E
A R L A D S R V K I A Q A V E K L E T Q L A D G R D W L M G D F S I A D L E T H A W L A G M R S I
V P E A F A A S P L T N A W E I R L R A R P A V A R A L G L A N V A H P E A I W A P G P E I N R W G

Cell-free synthesis of BaeB (Saro_2872) and BaeA (Saro_2873)

Plasmid for expressing BaeB

Plasmid pEU-NGFP (3) was amplified via PCR using primers "pEU-HiFi-ATW-R" and "pEU-HiFi-ATW-F" to generate a linear fragment in which the gene for Green Fluorescent Protein has been removed. pVP302K/Ntag-2872 was amplified via PCR using primers "Saro2872-pEU2394-HiFi-F" and "Saro2872-pEU2394-HiFi-R" to generate a linear fragment containing the coding sequence for the TEV protease recognition site followed by Saro_2872. These linear fragments were combined using the NEBuilder HiFi Assembly system to create a plasmid that was missing a short sequence upstream of the translational start site. To add this sequence, we amplified the plasmid using kinase phosphorylated primers "pEU-2872-fix-R" and "pEU-2872-fix-F". The linear fragment was circularized using T4 DNA ligase to form plasmid pEU-H2872, which contains a sequence for a His_6-tag, followed by a TEV protease recognition site, then Saro_2872.

The amino acid sequence of the resulting polypeptide is (with TEV protease recognition site underlined, and Met[1] of the native protein in bold):
M G H H H H H H A L A S E N L Y F Q S A I A G **M** S A L L Y H G E P N G A S L T V L A A L A E T G L
D I E C R R I D L L A G E R H S L P G I V D P V A L D L S I E G E G P V L V I D G E A M T E S V F L A Q
Y L D E A A G G V G L Q P T D A Y A R W E M M M W C R Q I T E R L S P A A A L L G N L A T S Q S A
I A A I P A E D F A I L A A R I V S D D L R E R W Q A L N D D A V N A A Q V A D S E T K V A A A V D
R C E K Q L G D G R E W L M G T F S I A D L V T Y S W L A G M E P L R P A A F A D A P L V K A W L
A R T A A R P C V Q A A L A R A T I S E P L R A W A P G P E I N R W G

Plasmid for expressing BaeA

Plasmid pEU-H2872 was amplified via PCR using primers "pEU-2872-fix-R" and "pEU2394 F" to generate a linear fragment in which the sequences for the His_6-tag, the TEV protease recognition site, and Saro_2872 were removed. *N. aromaticivorans* genomic DNA was amplified via PCR using primers "Saro_2873-pEU_HiFi-F" and "Saro_2873-pEU_HiFi-R" to generate a linear fragment containing Saro_2873 with ends that are complementary to the linear fragment generated directly above. These linear fragments were combined using the NEBuilder HiFi Assembly system to create plasmid pEU-2873.

Figure S1. HPLC traces of samples from assays of cell-free generated Saro_2873 (BaeA) and Saro_2872 (BaeB) polypeptides. Data are shown as absorbance units at 280 nm. (A) Racemic (β(*R*) and β(*S*)) MPHPV. (B) Racemic MPHPV + BaeA. (C) Racemic MPHPV + BaeB. (D) Racemic MPHPV + BaeA + BaeB. (E) Racemic MPHPV + BaeA + BaeB + NaLigE. (F) Racemic MPHPV + BaeA + BaeB + NaLigF1.

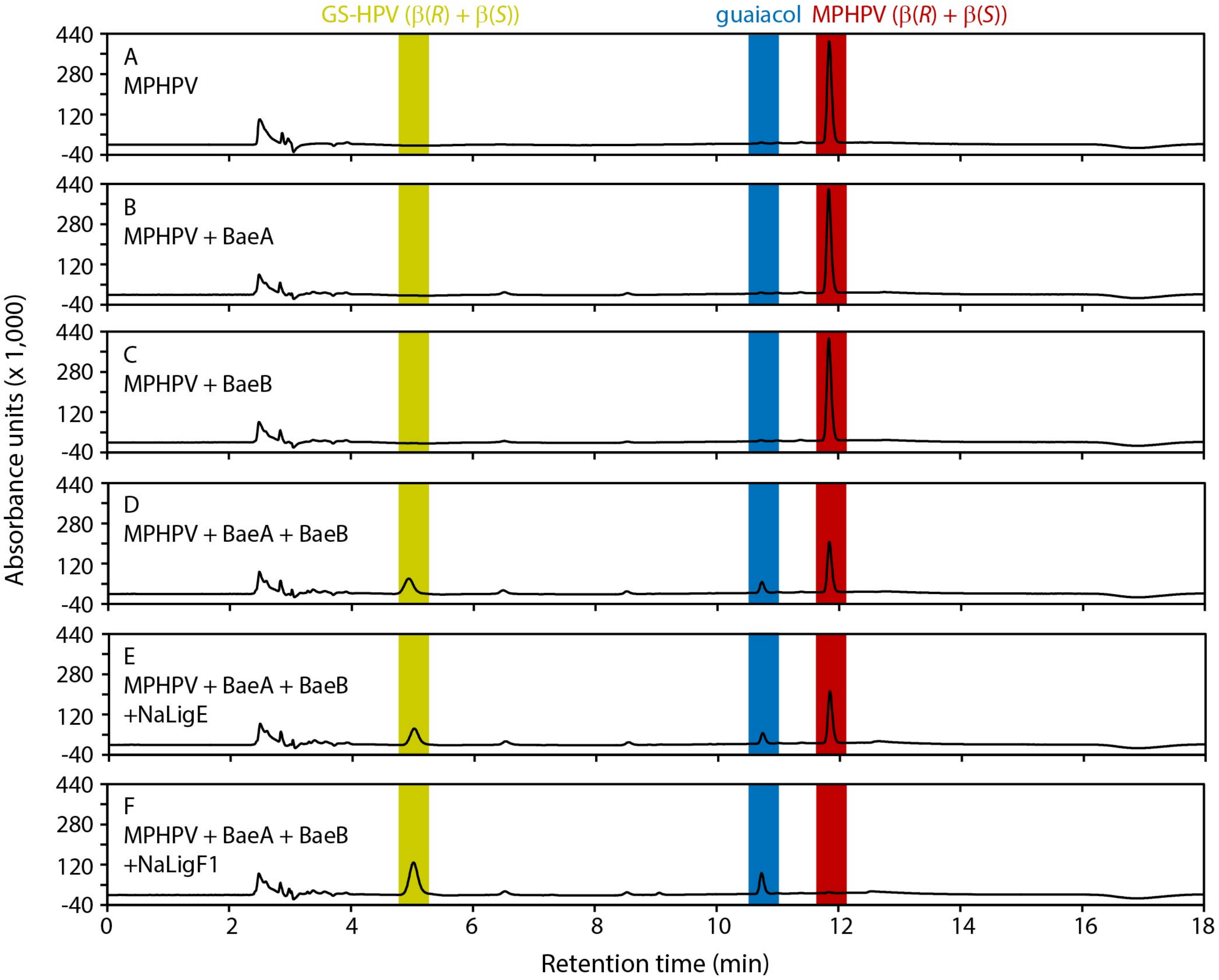

Figure S2. Genotypes of the *N. aromaticivorans* deletion mutants made in this study. Sequences were retrieved from GenBank (NC_007794.1). Intergenic regions use black font. The coding sequences of Saro_2405, Saro_2406, Saro_2871, Saro_2872, and Saro_2873 use red, purple, orange, green, and blue font, respectively. The 4 bp region of overlap between Saro_2873 and Saro_2872 uses brown font. Binding sites for primers used to delete genes are underlined. Regions shaded gray are missing in the respective deletion mutants. Start and stop codons are shown in uppercase letters. For the Saro_2872 deletion mutant, the C at position 3096845 was mutated to a T to eliminate the Saro_2872 start codon, while preserving the Saro_2873 stop codon.

Saro_2405 genomic sequence

```
2573461 tccatgtctc aacgcagaca gccgagtgag gggacggatg gaccctgaaa caagttcagg
2573521 gtgacggcaa ttgagtcaca aatttcgttt gtgttactaa caatatctgg cagactgctc
2573581 gcaacgacga gagaggattc ccATGgccgc caacaacacg atcactttct acgaccttgc
2573641 cctttcgacc ggcgcgacga tcagcccgtt cgtctgggcg acgaaatatg cgttgaagca
2573701 caagggcttc gacctcgacg tcgtgccagg cgggttcacc ggaattctcg aacgcaccgg
2573761 cggcaagacc gagcgccttc ccgcgatcgt cgacgacggc gagttcgttc tcgatagctg
2573821 gggcatcgtc gaatacctcg atgccaagta tccggaccgc cctgtcctga tcccgcacga
2573881 aagcgtcgcg gcgacgctca aggcgctcga caactggttc tggaatgctg ccgtcggacc
2573941 gtggatgttc tgcttctgcc aggactaccg cgatctctcg ctgccgcagg atcacgaata
2574001 cgtcacccac agccgcgaga agatgctcgg ccgcaagctc gaagaagtcc aggccggacg
2574061 cgaagagcgc ctgccgaaga tctccgccgc gctcgaaccg ctgcgcgccg cgcttgccca
2574121 gcaccagtgg ctcggcggct cctcgcccaa ctacgccgac taccgcatca tgggcggcat
2574181 cctgttcacc gcctcggtgt gcaagacgcc ggtgctcgcc aatgacgatc cattgcgcga
2574241 ctggatcgag cgctgcctcg acctctacgg cggcctgggc cgtcaccccg ggctgttccc
2574301 gctgttcggc ctggaacagc gcgaaggcga tcccgacctg ttcaatcgcg cggcaggcca
2574361 gggcggcatc tacaagcgca acaccggccc ggaatccacc cgcgccgaaa cccagcgcat
2574421 caccgaaggc atgaagaagT AAacgggtgg ggccggagcg atccggcccC TAtttcctga
```

Saro_2872 genomic sequence (opposite strand)

```
3096001 tggcgagacg ctgcgggtcg cggtcatgtc cgcggtcaaa gtagtcgatg gcgcgcattg
3096061 gTTAtcccca acggttgatt tcaggccccg gcgcccaggc gcggagcggt tcggaaatgg
3096121 tggcccgggc aagtgccgcc tgcacgcaag ggcgcgcggc ggtgcgggca agccaggcct
3096181 tgacaagcgg tgcatcggca aaggcggcag ggcggagcgg ctccatcccg gcaagccacg
3096241 agtaggtgac gagatcggcg atggagaaag tccccatcag ccattcgcgt ccatcgccaa
3096301 gctgcttctc gcagcggtcg acggcggcgg cgaccttggt ttcgctgtcg gcgacctgtg
3096361 cggcgttcac cgcatcgtcg ttcagggcct gccaccgctc gcgcaggtcg tcggaaacga
3096421 tccgtgcggc gagaatggcg aagtcctcgg ccgggatggc ggcgatggcg ctttgcgacg
3096481 tggcgagatt gccgagcagg gccgcggcgg gcgagaggcg ctcggtgatc tggcggcacc
3096541 acatcatcat ttcccagcgc gcataggcgt cggtcggctg gagccccacc ccgcccgccg
3096601 cctcgtccag atattgggcg aggaagacgg attcggtcat tgcttccccg tcgatcacca
3096661 gcaccggacc ttcgccttcg atggacaggt cgagcgcgac gggatcgacg atgccgggaa
3096721 gcgaatggcg ctcgcccgcc aggaggtcga tgcggcgaca ctcgatatcg aggcccgttt
3096781 ccgcaagcgc cgcaagaacg gtgagcgacg cgccgttggg ctcgccgtgg taaagaagcg
3096841 cgcTCATccc tcgatctccg ccatgatgag gccgtagcga tgggcaaggt ccgtcttgcc
```

Saro_2873 genomic sequence (opposite strand)

```
3096781 ccgcaagcgc cgcaagaacg gtgagcgacg cgccgttggg ctcgccgtgg taaagaagcg
3096841 cgcTCATccc tcgatctccg ccatgatgag gccgtagcga tgggcaaggt ccgtcttgcc
3096901 catggcccag gtcttcttca ccgcttcgcg ggtgtagaca cgcttgagcc agcgcatgat
3096961 gttcggcgtc ctgtccttgc ccgccagatc gggctgggaa atgggcagcg aataggtgct
3097021 gttgaagatg ttgatgtcgg ccaggctgta ctggttcgaa ccgacatagg gccgcttgcc
3097081 cagttcctct tccagcttgg cgatgcccag ccccacgcgg cggcggcttt cggccatctc
3097141 gctttccgag aagtcgccgt tgatcgcctt gcgccacgcg gtgcggcgtt cgggcaaggg
3097201 gatacggtcg atcgcggcgg caagttcggc ggggtcgcgc tggcggacca tgggaccgac
3097261 aaacacgctc cagccgatca tcgagaaact ggggccaagc cactggtcca tgaacttcat
3097321 ccaccagcgc acgcgccagc gatcctgcgc gtcggcgggc atgaggtccg gcccgtcgaa
3097381 gcggtcgttc acgtactcca tgatcgcggt gctttccgtc agcacctggc cattgtgcgt
3097441 catcgccggg atcgtgcctt gcgggttgat cgcaaggtat tccggcttgt gctgatcgaa
3097501 ctggagcatg tcgatgtaat ggctggaaaa gggcacgccc ttctccatca acgcgagcat
3097561 cggcttgcca gagttggcat tgggttccca atgatagagg cttacctcgt cCATccgctg
3097621 cccatccgct gcattgtctc tctcccgatt ccggtcagcg acggagtcgc gccggaattt
```

Figure S3. HPLC traces of samples from assays of recombinant BaeAB (Saro_2873-Saro_2872 complex). Data are shown as absorbance units at 280 nm. (A) Racemic (β(*R*) and β(*S*)) MPHPV. (B) Racemic MPHPV + NaLigE to generate enantiopure β(*S*)-MPHPV. (C) Racemic MPHPV + NaLigF1 to generate enantiopure β(*R*)-MPHPV. (D) β(*S*)-MPHPV + BaeAB. (E) β(*R*)-MPHPV + BaeAB.

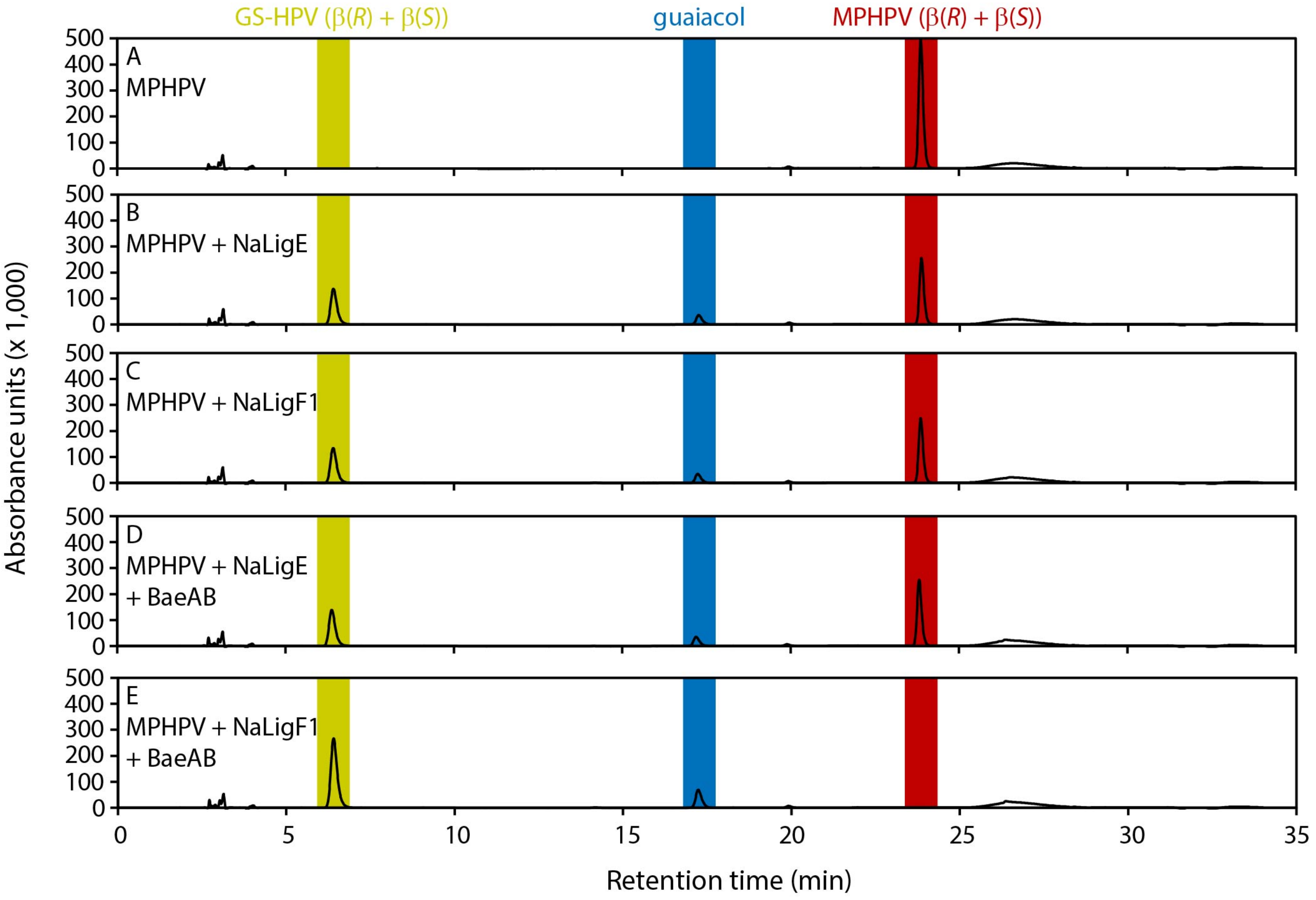

Figure S4: Full-length alignment of LigF homologues previously shown to cleave β(S)-MPHPV with BaeA (Saro_2873) and BaeB (Saro_2872) homologues from *N. aromaticivorans*, *Novosphingobium* sp. PP1Y, and *S. xenophagum*. Proteins and alignment method are the same as those in Fig. 6 in the main text. The serine residue (Ser^{14}) previously found to be involved in catalysis in LigF (4) is highlighted in green. The BaeA and BaeB residues substituted by alanine in the present study are highlighted in blue.

```
LigF          -MTLKLYSFGPGANSLKPLATLYEKGLEFEQVFVDPSKFEQHS------DWFKKINPRGQ  53
NaLigF1       --MLKLYSFGPAANSMKPLLTVFEKGLDVEKHRLDPAKFEHHT------DWFKAINPRGQ  52
LigF-NS       --MLTLYSFGPGANSLKPLLALYEKGLEFTPRFVDPTRFEHHE------EWFKKINPRGQ  52
MBE-GST4      --MLTLYSFGPGANSLKPLLALYEKGLEFTPRFVDPTKFEHHE------EWFKKINPRGQ  52
NaLigF2       -MALKYYHAEPLANSLKSMVPLKEKGLAYESIYVDLHKFEQHQ------PWFTAINPEGQ  53
BaeA          MDEVSLYHWEPNANSGKPMLALMEKGVPFSSHYIDMLQFDQHK------PEYLAINPQGT  54
PP1Y-BaeA     MAQVTLYHWEPNANSGKPMLTLMEKGVEFDSHYIDMLEFDQHR------PEYLAINPQGT  54
SxeBaeA       MTDVTLYHWEPNANSGKPMLALFEKGVAFDSHYLDLLNFDQHK------PDYLAVNPLGT  54
BaeB          -MSALLYHGEPNGASLTVLAALAETGLDIECRRIDLLAGERHSLPGIVDPVALDLSIEGE  59
PP1Y-BaeB     -MAMILYHGAPNGPSLIVLAALAETGVAVERRRIDLLAGDRHVLPGISEPVALNMGIEGE  59
SxeBaeB       -MTRILYHGQPNGPSFTVLAAAFEKDVTLDLREFDLVAGDRHA-PALPHPIEVDQSIEGE  58

LigF          VPALWHDGKVVTESTVICEYLEDVFPESGN---SLRPADPFKRAEMRVWTKWVDEYFCWC  110
NaLigF1       VPALVDGDKVVTESTVICEYLEDEYPTE-V---ALRPADSFGKAQMRIWTKWVDEYFCWC  108
LigF-NS       VPALDHDGHIITESTVICEYLEDAFPEA-P---RLRPVDPVMIAEMRVWTKWVDEYFCWC  108
MBE-GST4      VPALDHDGNVITESTVICEYLEDAFPDA-P---RLRPTDPVQIAEMRVWTKWVDEYFCWC  108
NaLigF2       VPVLDHDGTIITHTTVINEYLEDAFPDAQPADAPLRPRDPVGAARMRYWNKFIDEHVMNY  113
BaeA          IPAMTHNGQVLTESTAIMEYVNDRFDGP-----DLMPADAQDRWRVRWWMKFMDQWLGPS  109
PP1Y-BaeA     IPAMTHGSRVLVESTAIMEYVNEEFSGP-----DLMPKDALDRWRVRWWMKFMDQWLAPS  109
SxeBaeA       IPAMTHGQHVLTESTAIMEYVDEAFDGP-----RLMPVDPVDQWRTRWWMKFLDQWLAPS  109
BaeB          GPVLVIDGEAMTESVFLAQYLDEAAGGV-----GLQPTDAYARWEMMMWCRQITERLSPA  114
PP1Y-BaeB     GPVLVVDGEAMTDAVFLAQYFDELSEKA-----SLQPSDPYAHWEMMMWCRQITERLSPA  114
SxeBaeB       GPVFIVDGVAMTDSVFLACYLDEIGSGP-----ALRPADPYARWQMMAWCRYVIERVAPA  113

LigF          VSTIGWAFGIKAIAQKMSDEEFEEHINKNVPIPEQQLKWRRARNG-FPQEMLDEEFRKVG  169
NaLigF1       VSTIGWHRYVGNMVKSLSDAEFEEKV-KAIPVIEQQVKWRRAREG-FPQDMLDEEMRKIA  166
LigF-NS       VSTIGWERMIGPMARALSDEEFEAKV-ARIPVPEQRTKWRTARTG-FPKEVLDEEMRKIG  166
MBE-GST4      VSTIGWERGIGPMARALSDEEFEEKV-KRIPIPEQQAKWRSARAG-FPKEVLDEEMRKIR  166
NaLigF2       VSMHGWHRMVGVIARNIASGDFEKLL-ESIPLPDQRKKWATARSG-FSEADLANATAKIE  171
BaeA          FSMIGWSVFVGPMVRQRDPAELAAAI-DRIPLPERRTAWRKAINGDFSESEMAESRRRVG  168
PP1Y-BaeA     FSMIGWSVFVGPMVRQRDPAELEAAI-ERIPLPERRVSWRKAIHGTFSEAEIGESQRRVA  168
SxeBaeA       FSMIGWSVFVGPSVRQKDPAELEAAI-DRIPMPERRIAWRKAINGAFSAEEMAESQRRVA  168
BaeB          AALLGNLATSQSAIAAIPAEDFAILA-ARIVSDDLRERWQALNDDAVNAAQVADSETKVA  173
PP1Y-BaeB     AALLGNVEFSQETLGAMSDEQFEELT-DRIVSADLRSRWEILRDGAVDLAQITDSRTKVL  173
SxeBaeB       AACLGVAAHPPA-----------AVP-AGIASADLEQRWRDAVEGRADEARLADSRVKIA  161

LigF          VSVARLEETLSKQDYLVDTGYSLADICNFAIANGLQRPGGFFGDYVNQEKTPGLCAWLDR  229
NaLigF1       YSVRKLDDHLADHEWLVPGQYTLADICNFAIANGMQF---GFAELVNKQDTPHLVRWIEQ  223
LigF-NS       VSVNRLETRLAESPWLAGENFSLADVCNFAIANGMQN---GFSDIVNREATPHLVAWIEK  223
MBE-GST4      VSIDRLEKRLSESTWLAGEDYTLADICNFAIANGMEK---GFDDIVNTAATPNLVAWIER  223
NaLigF2       YALDKVEKQLGETKWLAGDTYTLADINFYSHCGAMVE--RMFPEMEVARRAPRLCEWRDR  229
BaeA          LGIAKLEEELGKRPYVGSNQYSLADINIFNSTYSLPI---SQPDLAGKDRTPNIMRWLKR  225
PP1Y-BaeA     LGIGMLEQELGKREWLASDAYSLADINGFNLAYAIPL---SQPSLCNDERTPNLLRWLRA  225
SxeBaeA       LGITYLEQALGQRDWLASNSYGLADINGFNLAYAMPL---SQPQLCNDDLTPNIMRWLRA  225
BaeB          AAVDRCEKQLGDGREWLMGTFSIADLVTYSWLAGMEP---LRPAA--FADAPLVKAWLAR  228
PP1Y-BaeB     QAVERCEQQLGDGREWLMGELTIADLTTFAWLAGMEQ---VLPES--FRDKPLTTAWLQR  228
SxeBaeB       QAVEKLETQLADGRDWLMGDFSIADLETHAWLAGMRS---IVPEA--FAASPLTNAWEIR  216

LigF          INARPAIKEMFEKSKREDLLKRQNEKVA------        257
NaLigF1       INERPAVKQMFAQVELEKLGPRE-----------        246
LigF-NS       INDRPACKAMFANSKSEFADRGQKVTA-------        250
MBE-GST4      INARPACIEMFAKSKSEFAARKPFAKSEEQAQA-        256
NaLigF2       VAARPAVAEALKSEDRTAPGLRVWSGEVR-----        258
BaeA          VYTREAVKKTWAMGKTDLAHRYGLI-MAEIEG--        256
PP1Y-BaeA     VYARPAVKKCWALGRTDMVKRVAIL-DGEQI---        255
SxeBaeA       IYARPATRACWALGRTDLARRISLL-ESEPA---        255
BaeB          TAARPCVQAALARATISEPLRA-WAPGPEINRWG        261
PP1Y-BaeB     VRSRPAVASALADGGA-RAATF-WAPGPEINRWG        260
SxeBaeB       LRARPAVARALGLANVAHPEAI-WAPGPEINRWG        249
```

Figure S5. Kinetics of breaking the β-aryl ether bond of β(*R*)-MPHPV by wild-type and variant BaeAB proteins. Reactions used 18 nM BaeAB, 23 nM BaeAB (B:S14A), 22 nM BaeAB (A:S15A), 24 nM BaeAB (A:S15A/ B:S14A), 98 nM BaeAB (A:N14A), or 70 nM NaLigE (all BaeAB concentrations are for the heterodimers; NaLigE concentration is for individual polypeptides of the homodimer). Curves are non-linear least squares best fits to the experimental data using the Michaelis-Menten equation.

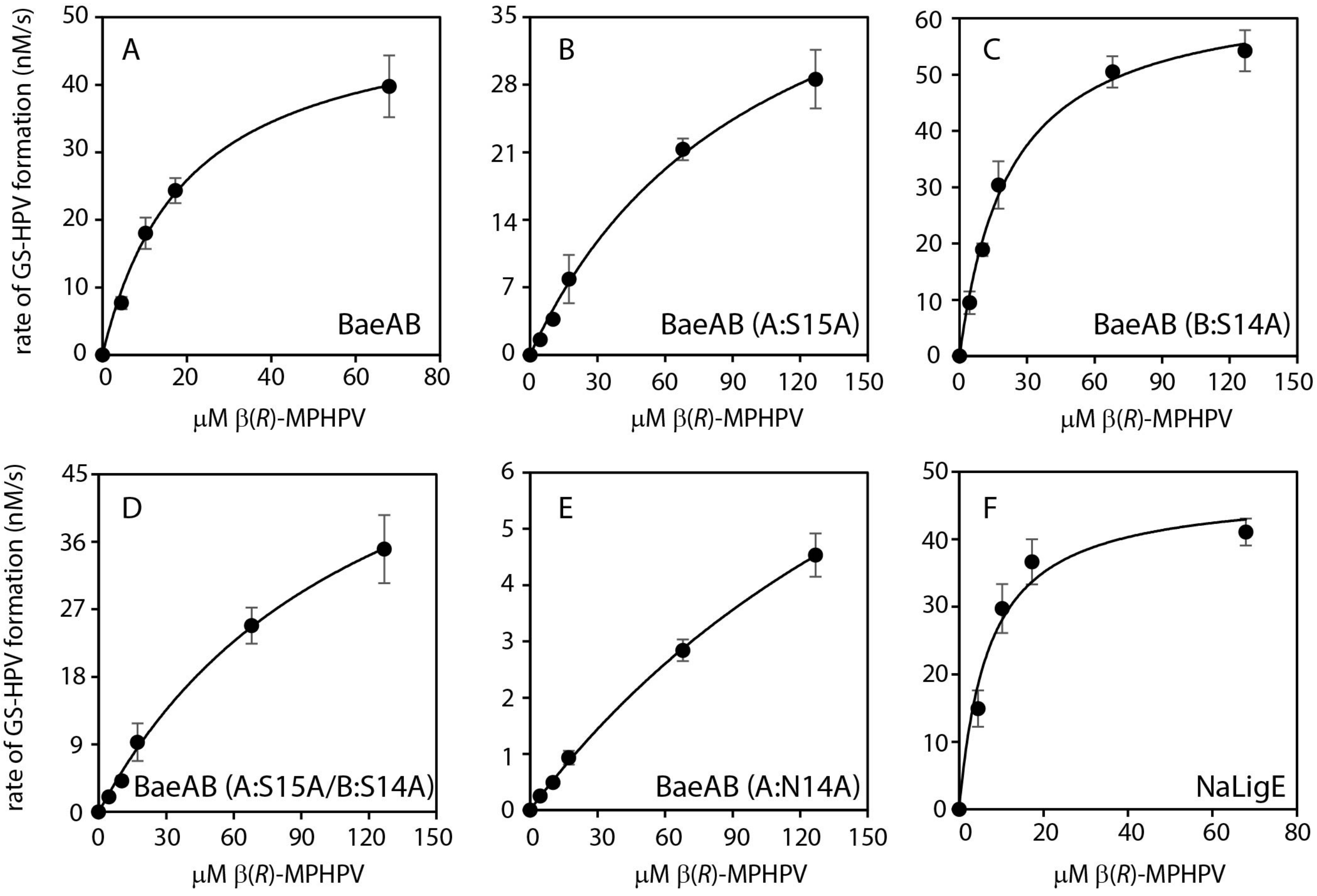

Figure S6: Percent amino acid sequence identity matrix for the known sphingomonad β-etherases. An alignment was made using Clustal Omega 1.2.4 involving the five sphingomonad proteins previously found to cleave the β(*S*)-aryl ether bond (LigF homologues; in green), the five sphingomonad proteins previously found to cleave the β(*R*)-aryl ether bond (LigE homologues; in orange), and the BaeA and BaeB subunits of the BaeAB homologues from *N. aromaticivorans*, *Novosphingobium* sp. PP1Y, and *S. xenophagum* found in this work to cleave β(*R*)-MPHPV. Proteins included are: NaLigE (Saro_2405; WP_011446047.1), NaLigF1 (Saro_2091; WP_041551020.1), NaLigF2 (Saro_2865; WP_052241956.1), BaeA (Saro_2873; WP_011446513.1), and BaeB (Saro_2872; WP_011446512.1) from *N. aromaticivorans* (2, 5); LigE (SLG_08660; WP_014075192.1), LigP (SLG_32600; WP_014077574.1), and LigF (SLG_08650; WP_014075191.1) from *Sphingobium* sp. SYK-6 (2, 5, 6); NsLigE (PP1Y_AT11664; WP_013832481.1), LigF-Ns (PP1Y_AT11660; WP_013832480.1), PP1Y-BaeA (PP1Y_AT11532; WP_013832467.1), and PP1Y-BaeB (PP1Y_AT11540; WP_013832468.1) from *Novosphingobium* sp. PP1Y (2, 5); MBE-GST5 (WP_039391125.1) and GST4 (WP_039391123.1) from *Novosphingobium* sp. MBES04 (7); and SxeBaeA (SX1_RS06450; WP_019052344.1) and SxeBaeB (SX1_RS06455; WP_019052345.1) from *S. xenophagum*.

	LigF	NaLigF1	LigF-NS	MBE-GST4	NaLigF2	BaeA	PP1Y-BaeA	SxeBaeA	BaeB	PP1Y-BaeB	SxeBaeB	LigE	LigP	NaLigE	NsLigE	MBE-GST5
LigF	100.0	60.6	64.4	66.9	39.4	35.6	34.0	32.8	23.4	24.3	24.1	19.7	17.6	18.5	18.8	20.5
NaLigF1	60.6	100.0	61.0	61.8	39.0	33.1	35.9	34.3	20.7	22.8	24.7	20.2	19.0	21.3	20.6	21.5
LigF-NS	64.4	61.0	100.0	83.2	42.0	35.1	35.5	37.9	24.8	24.9	25.1	21.2	20.4	21.8	19.8	21.2
MBE-GST4	66.9	61.8	83.2	100.0	44.1	34.4	36.1	38.5	25.4	26.3	24.1	21.0	20.0	21.3	20.3	20.7
NaLigF2	39.4	39.0	42.0	44.1	100.0	32.7	34.7	34.3	29.7	28.2	26.9	20.0	15.8	18.9	18.7	18.3
BaeA	35.6	33.1	35.1	34.4	32.7	100.0	72.9	69.8	23.8	24.3	23.7	16.7	16.0	16.0	15.5	15.9
PP1Y-BaeA	34.0	35.9	35.5	36.1	34.7	72.9	100.0	74.5	23.1	23.6	23.8	17.7	16.5	15.7	18.1	18.1
SxeBaeA	32.8	34.3	37.9	38.5	34.3	69.8	74.5	100.0	24.7	25.6	25.0	18.5	15.2	17.0	16.4	17.7
BaeB	23.4	20.7	24.8	25.4	29.7	23.8	23.1	24.7	100.0	65.0	53.8	17.5	16.1	14.8	18.1	16.4
PP1Y-BaeB	24.3	22.8	24.9	26.3	28.2	24.3	23.6	25.6	65.0	100.0	54.4	18.9	17.9	16.2	20.8	18.6
SxeBaeB	24.1	24.7	25.1	24.1	26.9	23.7	23.8	25.0	53.8	54.4	100.0	17.8	18.1	17.7	17.5	16.6
LigE	19.7	20.2	21.2	21.0	20.0	16.7	17.7	18.5	17.5	18.9	17.8	100.0	60.3	60.3	78.8	75.4
LigP	17.6	19.0	20.4	20.0	15.8	16.0	16.5	15.2	16.1	17.9	18.1	60.3	100.0	64.5	63.8	65.2
NaLigE	18.5	21.3	21.8	21.3	18.9	16.0	15.7	17.0	14.8	16.2	17.7	60.3	64.5	100.0	59.0	58.9
NsLigE	18.8	20.6	19.8	20.3	18.7	15.5	18.1	16.4	18.1	20.8	17.5	78.8	63.8	59.0	100.0	85.3
MBE-GST5	20.5	21.5	21.2	20.7	18.3	15.9	18.1	17.7	16.4	18.6	16.6	75.4	65.2	58.9	85.3	100.0

Figure S7: Amino acid sequence conservation amongst predicted BaeA and BaeB homologues. Percent amino acid sequence identities to BaeA and BaeB from *N. aromaticivorans* are shown for the 43 putative BaeAB homologues in Table S1. Values are from a BLASTp search of the NCBI non-redundant protein database. The three enzymes shown in this study to cleave β(*R*)-MPHPV are labeled with their species of origin.

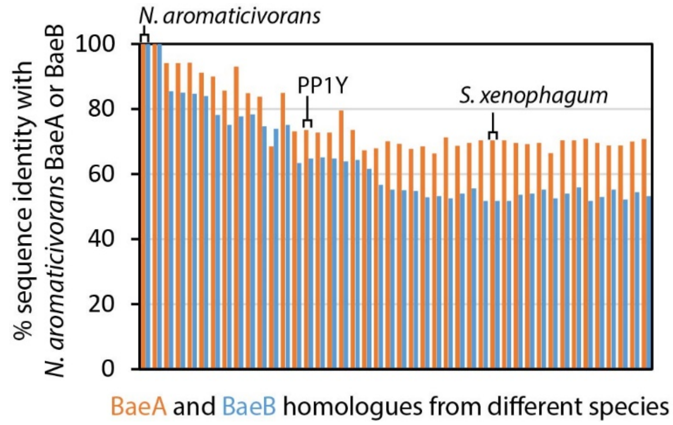

Figure S8. HPLC traces of samples from assays of recombinant *Novosphingobium* sp. PP1Y and *S. xenophagum* BaeAB homologues. Data are shown as absorbance units at 280 nm. (A) Racemic (β(*R*) and β(*S*)) MPHPV. (B) Racemic MPHPV + NaLigE to generate enantiopure β(*S*)-MPHPV. (C) β(*S*)-MPHPV + PP1Y-BaeAB. (D) β(*S*)-MPHPV + SxeBaeAB. (E) Racemic MPHPV + NaLigF1 to generate enantiopure β(*R*)-MPHPV. (F) β(*R*)-MPHPV + PP1Y-BaeAB. (G) β(*R*)-MPHPV + SxeBaeAB.

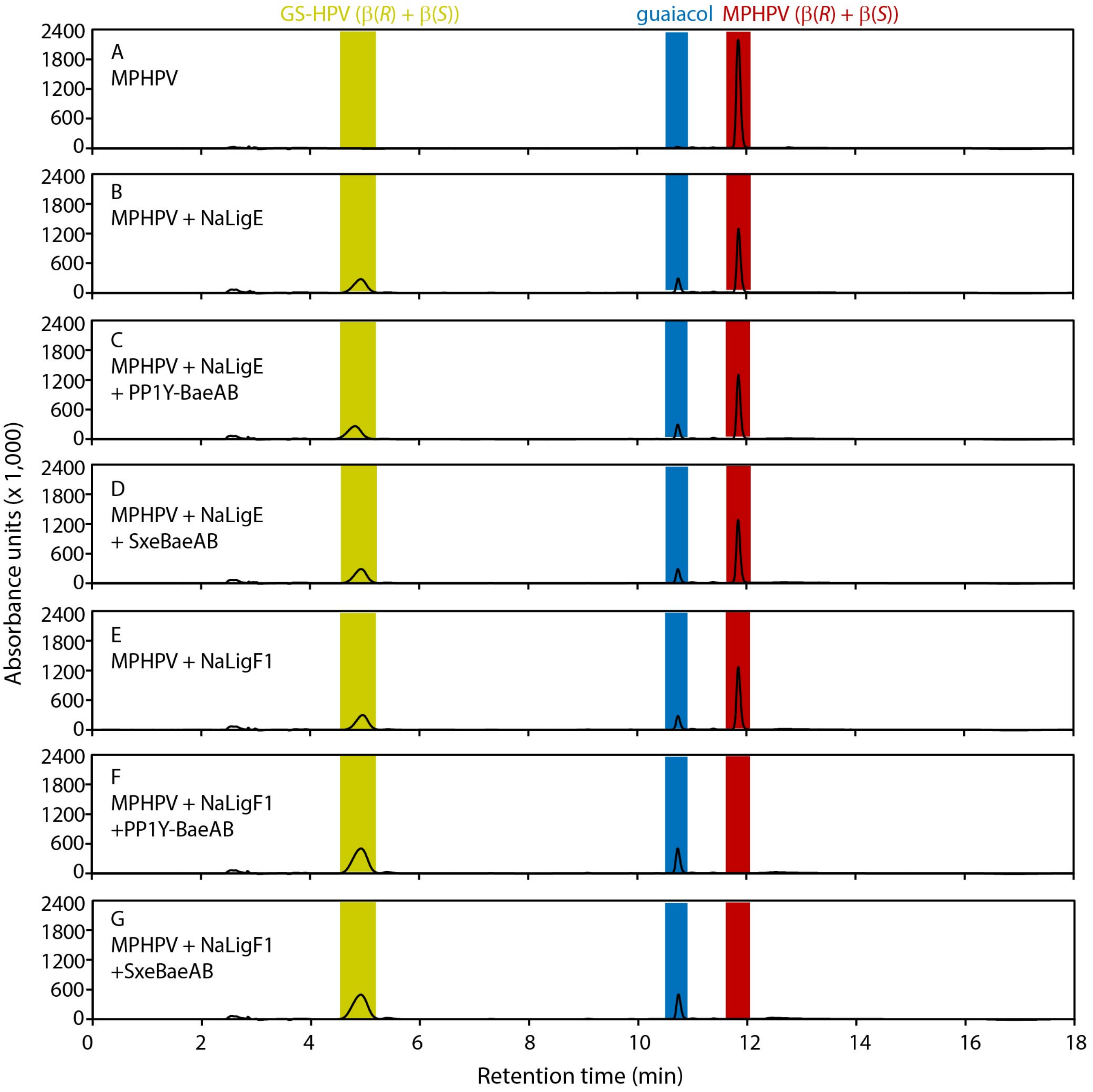

Figure S9. Compositions of the running buffer used for HPLC analysis of aromatic compounds. Percent methanol in the running buffer is shown. The remainder of the running buffer was an aqueous solution of 5 mM formic acid and 5% acetonitrile. The flow rate for both methods was 1 mL/min. (A) 34 min method used to analyze extracellular samples from growth experiments and some samples from *in vitro* enzyme reactions with recombinant BaeAB. (B) 18 min method used to analyze samples from *in vitro* reactions with cell-free generated polypeptides and recombinant BaeAB, and *in vitro* kinetics experiments.

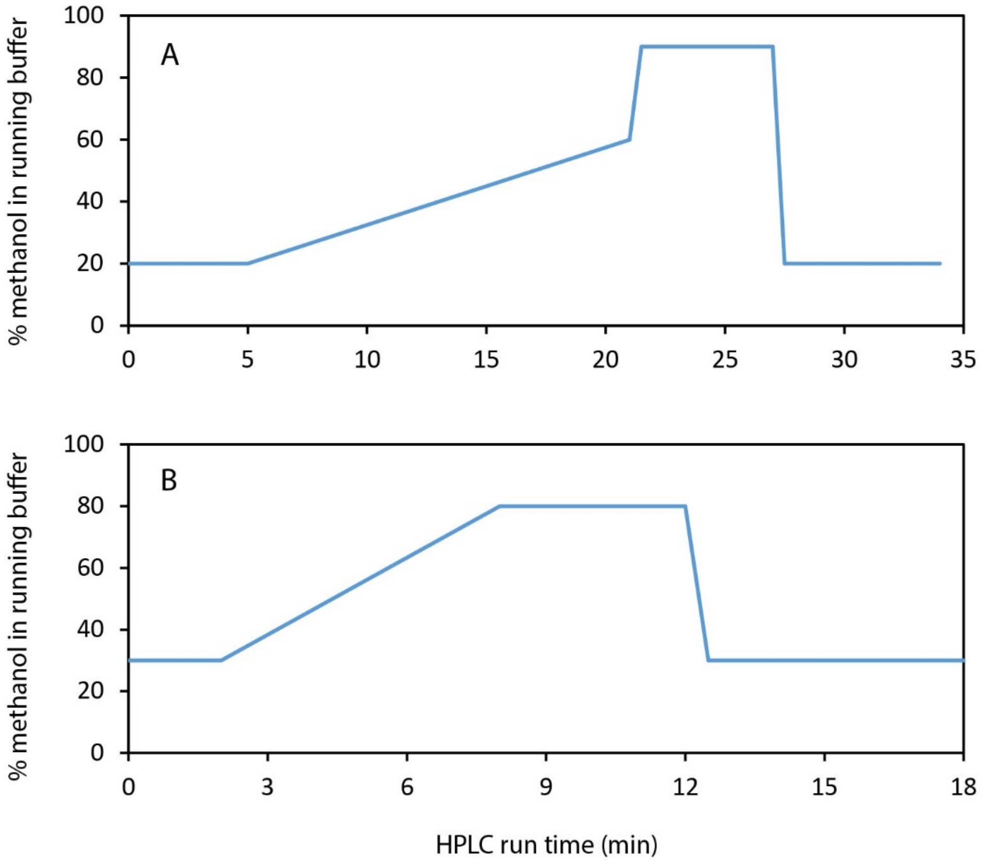

Table S1. Homologues of BaeA (Saro_2873), BaeB (Saro_2872), and NaLigE (Saro_2405). BLASTp searches were performed using the NCBI non-redundant protein database. The Overlap column shows how many base pairs the ORFs for the BaeA and BaeB proteins overlap by in each genome; for *Novosphingobium* sp. 225, there is 1 bp between the ORFs. The species shown are those that contain both a BaeA and BaeB homologue and/or a NaLigE homologue (criteria for homology are: amino acid sequence identity >50% and Bit score >200).

	**BaeA homologues**					**BaeB homologues**				**NaLigE homologues**			
**Species**	**Accession number**	**E-value**	**Bit Score**	**% identity**	**over lap**	**Accession number**	**E-value**	**Bit Score**	**% identity**	**Accession number**	**E-value**	**Bit Score**	**% identity**
Novosphingobium aromaticivorans DSM 12444	WP_011446513.1	0	531	100	4	WP_011446512.1	0	518	100	WP_011446047.1	0	579	100
Novosphingobium stygium	WP_011446513.1	0	531	100	4	WP_011446512.1	0	518	100	WP_011446047.1	0	579	100
Novosphingobium sp. CCH12-A3	WP_039331625.1	0	506	94.141	4	WP_062346619.1	1.09E-158	450	85.441	WP_062343074.1	0	551	94.624
Novosphingobium subterraneum	WP_039331625.1	0	506	94.141	4	WP_039331626.1	1.04E-157	447	85.057	WP_039335072.1	0	550	94.265
Novosphingobium sp. B1	WP_084279871.1	0	506	94.231	4	WP_084279870.1	4.32E-157	446	84.674	WP_084280065.1	0	556	95.699
Novosphingobium sp. 12-62-10	OYW48657.1	1.04E-175	493	91.154	4	OYW48658.1	8.71E-156	442	83.908	OYW49013.1	0	556	96.043
Novosphingobium sp. 32-60-15	OYX62555.1	8.53E-173	485	89.961	4	OYX62554.1	3.81E-148	423	78.161	OYX64790.1	0	558	95.341
Novosphingobium sp. PASSN1	OYU34959.1	1.72E-162	459	85.659	1	OYU34958.1	6.36E-139	400	75.096	OYU35660.1	0	545	94.265
Novosphingobium sp. AAP83	WP_054106620.1	5.82E-179	501	92.969	4	WP_054106619.1	1.42E-135	391	77.778	WP_054106368.1	0	553	94.624
Novosphingobium fuchskuhlense	WP_067907924.1	1.27E-160	454	84.884	1	WP_067907927.1	7.05E-134	387	78.378	WP_067913477.1	0	542	93.548
Novosphingobium sp. MD-1	GAO54711.1	1.76E-159	452	83.784	1	GAO54712.1	4.32E-126	368	74.721	GAO55515.1	0	543	93.502
Novosphingobium lentum	WP_084356031.1	2.71E-126	369	68.526	4	WP_068074747.1	4.23E-125	365	73.946				
Novosphingobium sp. SCN 66-18	ODU69497.1	6.08E-162	458	84.942	1	ODU69498.1	6.08E-124	362	75.092	ODU67585.1	0	544	93.502
Novosphingobium sp. MBES04	WP_039391102.1	1.40E-141	406	73.123	1	WP_039391104.1	1.03E-117	346	63.396	WP_039391125.1	9.47E-111	330	62.055
Novosphingobium sp. PP1Y	WP_013832467.1	8.08E-141	404	73.518	1	WP_013832468.1	8.67E-116	341	64.751	WP_013832481.1	2.74E-110	328	62.55
Novosphingobium mathurense	WP_079730288.1	1.06E-139	401	72.727	1	WP_079730289.1	4.19E-115	340	65.134	WP_079730390.1	7.91E-110	327	62.151
Novosphingobium sp. KN65.2	WP_054947720.1	9.40E-140	402	72.727	1	WP_054947721.1	1.21E-114	338	64.751	WP_054947729.1	4.96E-109	325	61.753
Erythrobacter sp. SG61-1L	WP_054532238.1	2.95E-150	428	79.528	4	WP_054531102.1	1.93E-112	333	63.846	WP_054530882.1	7.27E-163	462	79.928
										WP_054530343.1	3.46E-135	392	66.667
Novosphingobium sp. GV010	WP_110873420.1	3.62E-140	402	73.518	1	WP_110873419.1	8.38E-110	326	64.368	WP_110873471.1	3.45E-110	328	60.227
Novosphingobium sp. CF614	WP_091143995.1	9.78E-122	356	67.331	4	WP_091143994.1	2.49E-98	297	61.628				

Novosphingobium sp. B 225	WP_088311128.1	1.93E-124	363	67.843	-1	WP_088311129.1	1.64E-91	280	56.705	WP_088311142.1	4.85E-110	328	58.608
Sphingomonas hengshuiensis	WP_044331476.1	3.81E-128	372	70.04	4	WP_044331478.1	7.18E-86	265	55.172	WP_044331491.1	5.28E-97	295	59.289
Sphingomonas sp. L3B27	PVX29998.1	2.94E-127	370	69.231	4	PVX29997.1	5.18E-85	263	54.962				
Sphingorhabdus sp. M41	WP_067198469.1	5.77E-125	364	67.729	4	WP_067198471.1	8.56E-85	262	54.789				
Sphingomonadales bacterium CG_4_10_14_3_um_filter_58_15	PIX64599.1	2.91E-126	367	68.526	4	PIX64600.1	1.49E-84	262	52.852				
Sphingobium sp. 66-54	OJY66875.1	2.08E-123	360	66.27	7	OJY66876.1	1.54E-83	259	53.257	OJY67521.1<br>OJY68654.1	9.72E-136<br>2.00E-112	394<br>334	66.788<br>57.348
Marinicaulis flavus	WP_104831064.1	1.50E-134	388	71.2	2	WP_104831065.1	2.28E-83	259	52.49	WP_104830666.1	1.23E-156	446	78.102
Sphingomonadaceae bacterium KCTC 52780	WP_114953514.1	1.26E-131	381	68.651	1	WP_114953513.1	2.97E-83	259	54.023				
Sphingobium sp. Leaf26	WP_056685189.1	2.48E-131	380	69.565	4	WP_056685186.1	5.64E-83	258	55.556				
Sphingobium xenophagum	WP_019052344.1	2.61E-133	385	70.356	4	WP_019052345.1	7.83E-83	257	51.724				
Sphingobium sp. AEW4	WP_107124596.1	8.06E-133	384	70.356	4	WP_107124595.1	1.32E-82	257	51.724				
Sphingobium sp. AP50	WP_093083564.1	4.55E-132	382	69.565	4	WP_093083566.1	4.48E-81	253	53.64				
Sphingobium mali	WP_066818865.1	1.61E-126	368	69.2	4	WP_066818868.1	1.33E-80	252	54.023				
Sphingobium czechense	WP_066609195.1	1.85E-131	380	69.565	4	WP_066609194.1	2.71E-80	251	55.172				
Sphingobium wittichii	WP_037526943.1	1.21E-123	361	66.4	4	WP_037526918.1	3.67E-80	251	52.49				
Sphingobium sp. Z007	WP_088182824.1	2.03E-133	385	70.356	4	WP_088182825.1	4.28E-80	250	54.023				
Sphingomonas sp. 66-10	OJU17889.1	1.96E-129	375	70.4	4	OJU17890.1	6.69E-80	250	55.894				
Sphingomonas sp. Cra20	WP_100283326.1	4.50E-132	382	70.8	4	WP_100283325.1	8.98E-80	249	51.711				
Sphingomonas asaccharolytica	WP_066804573.1	1.48E-126	368	69.6	4	WP_066804576.1	1.03E-79	249	52.874				
Sphingobium sp. YR768	WP_093015917.1	3.85E-131	380	68.775	4	WP_093015914.1	1.99E-79	249	55.133				
Gammaproteobacteria bacterium 13_2_20CM_66_19	OLB14825.1	7.46E-127	369	68.8	4	OLB14824.1	1.86E-78	246	52.107				
Sphingobium sp. YG1	BBD02407.1	2.20E-131	380	69.96	4	BBD02406.1	2.64E-78	246	54.406				
Sphingobium sp. TCM1	WP_066863138.1	7.64E-134	387	70.751	4	WP_066863141.1	7.84E-78	244	53.257				
Novosphingobium sp. 35-62-5										OYX94051.1	0	558	95.324

Novosphingobium sp. NDB2Meth1			WP_072379148.1	0	525	89.964
Novosphingobium sp. AAP93			WP_054121822.1	0	522	89.606
Novosphingobium capsulatum			WP_062781915.1	8.74E-180	505	86.022
Novosphingobium sp. AAP1			WP_054131978.1	1.15E-179	504	86.022
Novosphingobium sp. GV064			WP_107715663.1	4.68E-179	503	85.663
Novosphingobium sp. GV055			WP_107715663.1	4.68E-179	503	85.663
Novosphingobium sp. GV061			WP_107715663.1	4.68E-179	503	85.663
Novosphingobium sp. GV079			WP_107715663.1	4.68E-179	503	85.663
Novosphingobium sp. GV027			WP_107715663.1	4.68E-179	503	85.663
Novosphingobium sp. B-7			WP_028657990.1	2.56E-178	501	85.663
Altererythrobacter sp. B11			BBC73886.1	6.60E-165	467	82.143
Sphingomonadales bacterium 63-6			OJW72302.1	4.02E-162	460	79.137
			OJW69813.1	2.24E-133	387	65.926
Altererythrobacter sp. 66-12			OJU60283.1	1.21E-159	454	80
Altererythrobacter sp. Root672			WP_055920889.1	2.44E-158	451	79.286
			WP_055921561.1	3.44E-124	364	62.409
Altererythrobacter atlanticus			WP_046903062.1	2.12E-157	448	78.214
			WP_046903179.1	7.81E-126	368	63.139
Sphingomonadales bacterium 12-68-11			OYW45403.1	2.32E-144	415	73.455
Sphingomonadales bacterium 32-64-17			OYX66856.1	4.19E-140	404	70.182
Sphingobium sp. SYK-6			WP_014077574.1	7.59E-135	391	65.934
			WP_014075192.1	2.13E-116	344	61.255
Novosphingobium sp. SYSU G00007			WP_109797409.1	1.42E-134	390	67.803
Sphingobium sp. SCN 64-10			ODT90752.1	2.46E-132	385	65.201
Novosphingobium sp. SCN 63-17			ODU84760.1	1.95E-116	345	60.364

Novosphingobium acidiphilum			WP_028641482.1	3.96E-109	326	59.636
Novosphingobium sp. ST904			WP_054436034.1	1.80E-108	324	60.853
Novosphingobium sp. FSW06-99			WP_067615430.1	1.36E-107	322	58.545
Novosphingobium sp. Fuku2-ISO-50			WP_067743869.1	2.26E-107	322	59.636

Table S2: **Primers used to modify the *N. aromaticivorans* genome and to create enzyme expression plasmids**

Name	Sequence	Notes
pK18-ligE OvExt F	5′-GTTTCTGCGGACTGGCTTTCTAGATGTTCCAGTGCTC TACAACCAGTCGTACCACATG-3′	Underlined region is complementary to pK18msB-MCS
pK18-ligE OvExt R	5′-CGATTCATTAATGCAGCTGGCACGACAGCGAGTTGA ACGAAACCTCCTCGTTCATG-3′	Underlined region is complementary to pK18msB-MCS
Saro2405 ligE del F	5′-GCATCACCGAAGGCATGAAGAAGTAAACG-3′	
Saro2405 ligE del R	5′-GTGACTCAATTGCCGTCACCCTGAACTTG-3′	
Saro_2872 ampl AseI F2	5′-CATCattaATTCGACCTGGCCATAGGACTG-3′	AseI site (underlined); lowercase bases do not match template
Saro_2872 ampl XbaI R	5′-taGttCtaGACCATCTTTTCCGCTGGAGC-3′	XbaI site (underlined); lowercase bases do not match template
Saro_2872 del R	5′-GCTTGTCAAGGCCTGGCTTGC-3′	
Saro_2872 del F	5′-TtATCCCTCGATCTCCGCCATGATGAG-3′	lowercase base does not match template
Saro_2873-pk18 hifi ampl R	5′-GTTTCTGCGGACTGGCTTTCTAGATGTTCCCTACAAG GGAGGGCAGTGAAATGAAGC-3′	Underlined region is complementary to pK18msB-MCS
Saro_2873 hifi del F	5′-CATCCCTCGATCTCGTCCATCCGCTGCCCATCC-3′	Underlined region is complementary to Saro_2873 hifi del R
Saro_2873-pk18 hifi ampl F	5′-CGATTCATTAATGCAGCTGGCACGACAGGGACGAAT GATAGACCAGCCACTTCAGG-3′	Underlined region is complementary to pK18msB-MCS
Saro_2873 hifi del R	5′-GATGGACGAGATCGAGGGATGAGCGCGCTTCTTTA CC-3′	Underlined region is complementary to Saro_2873 hifi del F
Saro2872 Ctag BsaI F	5′-GGCatctgcgaGaccTCCCCAACGGTTGATTTCAG-3′	BsaI site (underlined); lowercase bases do not match template
Saro2872 Ctag BspHI R	5′-CGAGtcATGAGCGCGCTTCTTTACCACG-3′	BspHI site (underlined); lowercase bases do not match template
pVP302K Ctag BsaI F	5′-CTGCGGTCTCGCAGATGGTAAAATTCTG-3′	BsaI site (underlined)
pVP302K Ctag NcoI R	5′-GGTGATGTCCCATGGTTAATTTCTCCTCTTTAATG-3′	NcoI site (underlined)
Ctag 2872-pVP add Stop R	5′-CGAGttaTCCCCAACGGTTGATTTCAGG-3′	lowercase bases do not match template
pVP302K Ntag HindIII F	5′-CATTAAaAGcTTAAACGAATTCGGACTCGGTACGC-3′	HindIII site (underlined); lowercase bases do not match template
2872-pVP C to Ntag F	5′-caagcgaaaatctgtattttcagagcgcgatcgcaggaATGAGCGCGCTTC TTTACCACG-3′	lowercase bases do not match template
pVP302 C to Ntag R	5′-ccaatgcatggtgatggtgatgatggtgatgtcccatGGTTAATTTCTCCTC TTTAATG-3′	lowercase bases do not match template
Saro2872 gNtag R	5′-caagcgaaaatctgtattttcagagcgcgatcgcaggaAGCGCGCTTCTTT ACCACGG-3′	lowercase bases do not match template
Saro2872 gNtag F	5′-ccaatgcatggtgatggtgatgatggtgatgtaTCATCCCTCGATCTCCG CCATGATG-3′	lowercase bases do not match template
2872-3_pVP_HiFi_F	5′-CTAACTTTGTTATTTTCGGCTTTCTGTTATCCCCAACG GTTGATTTCAGG-3′	Underlined region is complementary to pVP302K
Saro2872-3NOTAG_pVP_HiFi_R	5′-GAATTCATTAAAGAGGAGAAATTAACCATGGACGAG GTAAGCCTCTATCATTGG-3′	Underlined region is complementary to pVP302K
pVP302K-HiFi-noTag-R	5′-GGTTAATTTCTCCTCTTTAATGAATTCTGTGTGAAAT TG-3′	
pVP302K-HiFi-ATW-F	5′-CAGAAAGCCGAAAATAACAAAGTTAGCCTGAGCTG-3′	

Saro2872-3Ntag_pVP_HiFi_R	5′-GTATTTTCAGAGCGCGATCGCAGGAATGGACGAGGTAAGCCTCTATCATTGG-3′	Underlined region is complementary to pVP302K
pVP302K-HiFi-ATW-R	5′-TCCTGCGATCGCGCTCTGAAAATACAGATTTTCG-3′	
Saro2872-S14A_R	5′-CGCGgCGCTCACCGTTCTTGC-3′	Lowercase g introduces S→A mutation in underlined codon
Saro2872-S14A_F	5′-CCGTTGGGCTCGCCGTGGTAAAGAAG-3′	
Saro2873-S15A_R	5′-GCAAGCCGATGCTCGCGTTGATG-3′	
Saro2873-S15A_F	5′-CAGcGTTGGCATTGGGTTCCCAATGATAGAG-3′	Lowercase c introduces S→A mutation in underlined codon
Saro2873-N14A_F	5′-CAGAGgcGGCATTGGGTTCCCAATGATAGAG-3′	Lowercase gc introduces N→A mutation in underlined codon
PP1Y_BaeE_Ntag_pVP_HiFi_F	5′-GTATTTTCAGAGCGCGATCGCAGGAATGGCGCAAGTGACACTGTACCACTG-3′	Underlined region is complementary to pVP302K
PP1Y_BaeE_Ntag_pVP_HiFi_R	5′-CTAACTTTGTTATTTTCGGCTTTCTGGGTCATCCCCAGCGGTTGATCTCC-3′	Underlined region is complementary to pVP302K
Sxeno-BaeE_Ntag_pVP_HiFi_F	5′-GTATTTTCAGAGCGCGATCGCAGGAATGACCGACGTTACCCTCTACCATTGG-3′	Underlined region is complementary to pVP302K
Sxeno-BaeE_Ntag_pVP_HiFi_R	5′-CTAACTTTGTTATTTTCGGCTTTCTGTCAGCCCCAGCGGTTAATCTCC-3′	Underlined region is complementary to pVP302K
pEU-HiFi-ATW-R	5′-GTGATGATGATGATGATGTCCCATTAAC-3′	
pEU-HiFi-ATW-F	5′-TAGTTTAAACGAATTCGAGCTCGG-3′	
Saro2872-pEU2394-HiFi-F	5′-GGACATCATCATCATCATCACGCATTGGCAAGCGAAAATCTGTATTTTCAG-3′	Underlined region is complementary to pEU
Saro2872-pEU2394-HiFi-R	5′-CCGAGCTCGAATTCGTTTAAACTACGAGTTATCCCCAACGGTTGATTTCAGG-3′	Underlined region is complementary to pEU
pEU-2872-fix-R	5′-CATTAACTAACTAGTGTAGTTGTAGAATGTAAAATGTAATGTTGTTGTTGTTTG-3′	Underlined region was missing in originally created pEU-2872
pEU-2872-fix-F	5′-GGACATCATCATCATCATCACGCATTGG-3′	
Saro_2873-pEU_HiFi-F	5′-CAACTACACTAGTTAGTTAATGGACGAGGTAAGCCTCTATCATTGG-3′	Underlined region is complementary to pEU
Saro_2873-pEU_HiFi-R	5′-CGAGCTCGAATTCGTTTAAACTACTCATCCCTCGATCTCCGCCATG-3′	Underlined region is complementary to pEU
pEU2394 F	5′-GTAGTTTAAACGAATTCGAGCTCGGTACC-3′	

Table S3: Proteins used in the phylogenetic tree in Fig. 7

WP_011446047.1 MULTISPECIES: beta-1,3-glucanase [Novosphingobium]	WP_066609183.1 glutathione S-transferase family protein [Sphingobium czechense]
OYW49013.1 1,3-beta-glucanase [Novosphingobium sp. 12-62-10]	WP_066804606.1 glutathione S-transferase family protein [Sphingomonas asaccharolytica]
WP_118074161.1 glutathione S-transferase family protein [Novosphingobium sp. THN1]	WP_093018627.1 glutathione S-transferase family protein [Sphingobium sp. YR768]
OYU35660.1 1,3-beta-glucanase [Novosphingobium sp. PASSN1]	WP_066854755.1 glutathione S-transferase family protein [Sphingobium sp. TCM1]
WP_067913477.1 1,3-beta-glucanase [Novosphingobium fuchskuhlense]	WP_088182463.1 glutathione S-transferase family protein [Sphingobium sp. Z007]
WP_054121822.1 1,3-beta-glucanase [Novosphingobium sp. AAP93]	OGT82223.1 hypothetical protein A3H91_17845 [Gammaproteobacteria bacterium RIFCSPLOWO2_02_FULL_61_13]
WP_107715663.1 MULTISPECIES: 1,3-beta-glucanase [Novosphingobium]	WP_051280663.1 glutathione S-transferase family protein [Novosphingobium acidiphilum]
WP_054530882.1 1,3-beta-glucanase [Erythrobacter sp. SG61-1L]	PP1Y_AT11660_LigF_WP_013832480.1
WP_055920889.1 1,3-beta-glucanase [Altererythrobacter sp. Root672]	Ga0077677_1041177-MBES04LigF_GST4_WP_039391123.1
OYW45403.1 1,3-beta-glucanase [Sphingomonadales bacterium 12-68-11]	SLG_08650_Syk6LigF_WP_014075191.1
OJY67521.1 1,3-beta-glucanase [Sphingobium sp. 66-54]	WP_011446513.1 MULTISPECIES: glutathione S-transferase family protein [Novosphingobium]
WP_109797409.1 1,3-beta-glucanase [Novosphingobium sp. SYSU G00007]	WP_039331625.1 MULTISPECIES: glutathione S-transferase family protein [Novosphingobium]
WP_046903179.1 1,3-beta-glucanase [Altererythrobacter atlanticus]	OYZ35984.1 glutathione S-transferase, partial [Novosphingobium sp. 16-62-11]
OJY68654.1 1,3-beta-glucanase [Sphingobium sp. 66-54]	ODU69497.1 glutathione S-transferase [Novosphingobium sp. SCN 66-18]
WP_110873471.1 glutathione S-transferase family protein [Novosphingobium sp. GV010]	WP_054532238.1 glutathione S-transferase family protein [Erythrobacter sp. SG61-1L]
WP_028641482.1 1,3-beta-glucanase [Novosphingobium acidiphilum]	WP_110873420.1 glutathione S-transferase family protein [Novosphingobium sp. GV010]
WP_054436034.1 glutathione S-transferase family protein [Novosphingobium sp. ST904]	WP_079730288.1 glutathione S-transferase family protein [Novosphingobium mathurense]
WP_044331491.1 glutathione S-transferase family protein [Sphingomonas hengshuiensis]	WP_088182824.1 glutathione S-transferase family protein [Sphingobium sp. Z007]
WP_083762546.1 glutathione S-transferase family protein [Parvibaculum lavamentivorans]	WP_107124596.1 glutathione S-transferase family protein [Sphingobium sp. AEW4]
WP_096333581.1 glutathione S-transferase family protein [Nannocystis exedens]	WP_114953514.1 glutathione S-transferase family protein [Sphingomonadaceae bacterium KCTC 52780]
WP_018697888.1 glutathione S-transferase family protein [Amorphus coralli]	WP_056685189.1 glutathione S-transferase family protein [Sphingobium sp. Leaf26]
KYF62888.1 hypothetical protein BE11_07400 [Sorangium cellulosum]	OJU17889.1 glutathione S-transferase [Sphingomonas sp. 66-10]
WP_095800044.1 glutathione S-transferase family protein [Mesorhizobium sp. WSM3866]	OLB14825.1 glutathione S-transferase [Gammaproteobacteria bacterium 13_2_20CM_66_19]
WP_109794066.1 glutathione S-transferase family protein [Rhizobiales bacterium]	WP_084356031.1 glutathione S-transferase family protein [Novosphingobium lentum]
WP_095823615.1 glutathione S-transferase family protein [Mesorhizobium sp. WSM3882]	WP_088311128.1 glutathione S-transferase family protein [Novosphingobium sp. B 225]
SLG_32600-Syk6LigP_WP_014077574.1	OJY66875.1 glutathione S-transferase [Sphingobium sp. 66-54]
PP1Y_AT11664-LigE_WP_013832481.1	PZN34065.1 glutathione S-transferase family protein [Proteobacteria bacterium]
SLG_08660-Syk6LigE_WP_014075192.1	WP_110872578.1 glutathione S-transferase family protein [Novosphingobium sp. GV010]
Ga0077677_1041178-MBES04LigE_GST5_WP_039391125.1	WP_054529228.1 glutathione S-transferase family protein [Erythrobacter sp. SG61-1L]
RPA4340_RpalustrisLigE_WP_027276361.1	WP_119082781.1 glutathione S-transferase family protein [Altererythrobacter sp. B11]
WP_012238501.1_Sorangium-cellulosum-LigE	PZN79012.1 protein ligF [Proteobacteria bacterium]
XP_007363869.1_Ds-GST1	WP_014075049.1 glutathione S-transferase family protein [Sphingobium sp. SYK-6]

ABD27301.1 glutathione S-transferase-like protein [Novosphingobium aromaticivorans DSM 12444]	WP_054947728.1 MULTISPECIES: glutathione S-transferase family protein [Novosphingobium]
WP_084279863.1 glutathione S-transferase family protein [Novosphingobium sp. B1]	WP_011446512.1 MULTISPECIES: glutathione S-transferase family protein [Novosphingobium]
WP_052241956.1 MULTISPECIES: glutathione S-transferase family protein [Novosphingobium]	WP_039331626.1 glutathione S-transferase [Novosphingobium subterraneum]
OYU33440.1 protein ligF [Novosphingobium sp. PASSN1]	OYX62554.1 glutathione S-transferase [Novosphingobium sp. 32-60-15]
WP_068074729.1 glutathione S-transferase family protein [Novosphingobium lentum]	WP_067907927.1 glutathione S-transferase family protein [Novosphingobium fuchskuhlense]
WP_082697614.1 glutathione S-transferase family protein [Novosphingobium fuchskuhlense]	ODU69498.1 glutathione S-transferase [Novosphingobium sp. SCN 66-18]
RJT21212.1 glutathione S-transferase family protein [Sphingomonadaceae bacterium KCTC 52944]	WP_079730289.1 glutathione S-transferase family protein [Novosphingobium mathurense]
WP_054132647.1 glutathione S-transferase family protein [Novosphingobium sp. AAP1]	WP_110873419.1 glutathione S-transferase family protein [Novosphingobium sp. GV010]
WP_062787547.1 glutathione S-transferase family protein [Novosphingobium capsulatum]	WP_088311129.1 glutathione S-transferase [Novosphingobium sp. B 225]
OJY66881.1 hypothetical protein BGP16_16740 [Sphingobium sp. 66-54]	WP_116469414.1 glutathione S-transferase [Sphingomonas sp. L3B27]
WP_114954118.1 glutathione S-transferase family protein [Sphingomonadaceae bacterium KCTC 52780]	OJY66876.1 hypothetical protein BGP16_16710 [Sphingobium sp. 66-54]
WP_066818877.1 glutathione S-transferase family protein [Sphingomonas mali]	WP_056685186.1 glutathione S-transferase family protein [Sphingobium sp. Leaf26]
WP_084238447.1 glutathione S-transferase family protein [Sphingomonas asaccharolytica]	WP_107124595.1 glutathione S-transferase [Sphingobium sp. AEW4]
PIX64606.1 protein ligF [Sphingomonadales bacterium CG_4_10_14_3_um_filter_58_15]	WP_066609194.1 glutathione S-transferase family protein [Sphingobium czechense]
SFG44676.1 glutathione S-transferase [Novosphingobium sp. CF614]	OJU17890.1 glutathione S-transferase [Sphingomonas sp. 66-10]
WP_110872575.1 glutathione S-transferase family protein [Novosphingobium sp. GV010]	WP_093015914.1 glutathione S-transferase [Sphingobium sp. YR768]
WP_082703412.1 glutathione S-transferase family protein [Novosphingobium sp. Fuku2-ISO-50]	WP_066863141.1 glutathione S-transferase family protein [Sphingobium sp. TCM1]
PZN34063.1 protein ligF [Proteobacteria bacterium]	PZN34066.1 hypothetical protein DIU71_03540 [Proteobacteria bacterium]
WP_116469409.1 glutathione S-transferase family protein [Sphingomonas sp. L3B27]	KPL66914.1 hypothetical protein SZ64_01660 [Erythrobacter sp. SG61-1L]
WP_066818898.1 glutathione S-transferase family protein [Sphingomonas mali]	OJY71178.1 hypothetical protein BGP16_11330 [Sphingobium sp. 66-54]
WP_019052363.1 glutathione S-transferase family protein [Sphingobium xenophagum]	PZN81421.1 hypothetical protein DIU56_00280 [Proteobacteria bacterium]
WP_093081587.1 glutathione S-transferase family protein [Sphingobium sp. AP50]	OJU58736.1 hypothetical protein BGO08_00255 [Altererythrobacter sp. 66-12]
RJT26469.1 glutathione S-transferase family protein [Sphingomonadaceae bacterium KCTC 52944]	WP_054106714.1 glutathione S-transferase family protein [Novosphingobium sp. AAP83]
WP_100283311.1 glutathione S-transferase family protein [Sphingomonas sp. Cra20]	OJW75869.1 hypothetical protein BGO57_16550 [Sphingomonadales bacterium 63-6]
WP_041551020.1 MULTISPECIES: glutathione S-transferase family protein [Novosphingobium]	WP_081912041.1 glutathione S-transferase family protein [Sphingomonas wittichii]
WP_082734789.1 glutathione S-transferase family protein [Novosphingobium sp. CCH12-A3]	WP_013832467.1_NovosphingobiumPP1Y-BaeA
SMC30538.1 glutathione S-transferase [Novosphingobium sp. B1]	WP_013832468.1_NovosphingobiumPP1Y-BaeB
OYX94961.1 protein ligF [Novosphingobium sp. 35-62-5]	WP_019052344.1_Sxenophagum-BaeA
WP_107715567.1 MULTISPECIES: glutathione S-transferase family protein [Novosphingobium]	WP_019052345.1_Sxenophagum-BaeB
WP_072381855.1 glutathione S-transferase family protein [Novosphingobium sp. NDB2Meth1]	Saro_2595
WP_067910024.1 glutathione S-transferase family protein [Novosphingobium fuchskuhlense]	SLG_04120
GAO55900.1 glutathione S-transferase [Novosphingobium sp. MD-1]	E.coli_YghU

OJU59935.1 protein ligF [Altererythrobacter sp. 66-12]	MBES04_GST3
WP_055919008.1 glutathione S-transferase family protein [Altererythrobacter sp. Root672]	E.coli_YfcG
WP_068075965.1 glutathione S-transferase family protein [Novosphingobium lentum]	SYK6-LigG_WP_041392591.1
WP_114951303.1 glutathione S-transferase family protein [Sphingomonadaceae bacterium KCTC 52780]	PP1Y-LigG_WP_041558818.1
WP_107124580.1 glutathione S-transferase family protein [Sphingobium sp. AEW4]	Tdenitrif-LigG_Tbd_1050; WP_011311562.1
WP_104831261.1 glutathione S-transferase family protein [Marinicaulis flavus]	GAM05532.1_MBES04-LigG

Supporting Information References

1. Kontur, W.S., C.A. Bingman, C.N. Olmsted, D.R. Wassarman, A. Ulbrich, D.L. Gall, R.W. Smith, L.M. Yusko, B.G. Fox, D.R. Noguera, J.J. Coon, and T.J. Donohue. 2018. Novosphingobium aromaticivorans uses a Nu-class glutathione S-transferase as a glutathione lyase in breaking the β-aryl ether bond of lignin. J. Biol. Chem. 293: 4955–4968.

2. Gall, D.L., J. Ralph, T.J. Donohue, and D.R. Noguera. 2014. A group of sequence-related sphingomonad enzymes catalyzes cleavage of β-aryl ether linkages in lignin β-guaiacyl and β-syringyl ether dimers. Environ. Sci. Technol. 48: 12454–12463.

3. Goren, M.A., A. Nozawa, S. Makino, R.L. Wrobel, and B.G. Fox. 2009. Cell-free translation of integral membrane proteins into unilamelar liposomes. Meth. Enzymol. 463: 647–673.

4. Helmich, K.E., J.H. Pereira, D.L. Gall, R.A. Heins, R.P. McAndrew, C. Bingman, K. Deng, K.C. Holland, D.R. Noguera, B.A. Simmons, K.L. Sale, J. Ralph, T.J. Donohue, P.D. Adams, and G.N. Phillips. 2016. Structural Basis of Stereospecificity in the Bacterial Enzymatic Cleavage of β-Aryl Ether Bonds in Lignin. J Biol Chem. 291: 5234–5246.

5. Picart, P., C. Müller, J. Mottweiler, L. Wiermans, C. Bolm, P. Domínguez de María, and A. Schallmey. 2014. From gene towards selective biomass valorization: bacterial β-etherases with catalytic activity on lignin-like polymers. ChemSusChem. 7: 3164–3171.

6. Masai, E., A. Ichimura, Y. Sato, K. Miyauchi, Y. Katayama, and M. Fukuda. 2003. Roles of the enantioselective glutathione S-transferases in cleavage of beta-aryl ether. J. Bacteriol. 185: 1768–1775.

7. Ohta, Y., S. Nishi, R. Hasegawa, and Y. Hatada. 2015. Combination of six enzymes of a marine Novosphingobium converts the stereoisomers of β-O-4 lignin model dimers into the respective monomers. Sci Rep. 5: 15105.
